# A Dissenters’ View on AppleSnail Immunobiology

**DOI:** 10.3389/fimmu.2022.879122

**Published:** 2022-05-26

**Authors:** Cristian Rodriguez, Israel A. Vega, Alfredo Castro-Vazquez

**Affiliations:** ^1^ IHEM, CONICET, Universidad Nacional de Cuyo, Mendoza, Argentina; ^2^ Departamento de Biología, Facultad de Ciencias Exactas y Naturales, Universidad Nacional de Cuyo, Mendoza, Argentina; ^3^ Instituto de Fisiología, Facultad de Ciencias Médicas, Universidad Nacional de Cuyo, Mendoza, Argentina

**Keywords:** *Biomphalaria*, hematopoiesis, hemocyte, nodulation, *Pomacea*, rhogocyte, hemocyanin, reno-pericardial complex

## Abstract

We stand as dissenters against the acceptance of scientific knowledge that has not been built on empirical data. With this in mind, this review synthesizes selected aspects of the immunobiology of gastropods and of apple snails (Ampullariidae) in particular, from morphological to molecular and “omics” studies. Our trip went through more than two centuries of history and was guided by an evo-devo hypothesis: that the gastropod immune system originally developed in the mesenchymal connective tissue of the reno-pericardial complex, and that in that tissue some cells differentiated into hematopoietically committed progenitor cells that integrate constitutive hemocyte aggregations in the reno-pericardial territory, whether concentrated in the pericardium or the kidney in a species-specific manner. However, some of them may be freed from those aggregations, circulate in the blood, and form distant contingent aggregations anywhere in the body, but always in response to intruders (i.e., pathogens or any other immune challenge). After that, we reviewed the incipient immunology of the Ampullariidae by critically revising the findings in *Pomacea canaliculata* and *Marisa cornuarietis*, the only ampullariid species that have been studied in this respect, and we attempted to identify the effectors and the processes in which they are involved. Particularly for *P. canaliculata*, which is by far the most studied species, we ask which hemocytes are involved, in which tissues or organs are integrated, and what cellular reactions to intruders this species has in common with other animals. Furthermore, we wondered what humoral factors could also integrate its internal defense system. Among the cellular defenses, we give an outstanding position to the generation of hemocyte nodules, which seems to be an important process for these snails, serving the isolation and elimination of intruders. Finally, we discuss hematopoiesis in apple snails. There have been contrasting views about some of these aspects, but we envision a hematopoietic system centered in the constitutive hemocyte islets in the ampullariid kidney.

## 1 Introduction

There is a paucity of information regarding the defense system of Ampullariidae (apple snails), and we will provide here a synthetic view on this subject. We may remark that synthesis is not a summary but an interconnected whole, in which gross morphology, histology, ultrastructure, and molecules are integrated to understand biological issues, not only at the level of the cell or the individual but at the wonderful scenario of evolution.

The synthetic look at apple-snail immunobiology we are here offering is presented as a sprout of the earliest history of the field, and of biology itself, when it was still a harsh and uncultivated territory. And this new look wants to incorporate the knowledge progressively acquired about this interesting gastropod family, but also selected aspects studied in other gastropods were included if they provided some immediate context to the growing immunobiology of the Ampullariidae (apple snails). We will honestly attempt to include here the currently accepted knowledge, our current opinions as well as the opinions of those that dissent with us. Because, after all, who can say who is the dissenter, and who is the holder of the accepted knowledge?

## 2 Lamarck, Biology as an Emergent Discipline, and the Dawn of Gastropod Immunobiology

Biology as an independent discipline with a unifying theory (evolution) came of age in the writings of Lamarck between 1799 and 1815 (1). Only seven years later, he published the last volume of *Histoire naturelle des animaux sans vertèbres* (2). There, Lamarck recognized *les Ampullaires* (i.e., the Ampullariidae, the apple snails) as a distinct taxon, separated from today’s Helicoidea and Lymnaeoidea^1^, which include garden and pond snails, respectively. That comes to our story because the study of gastropod immunity (traditionally understood as the reaction to intruders, i.e., pathogens or any other immune challenge) began with representatives of both superfamilies, while the Ampullariidae, a taxon that later entered the scene, were also defined in that publication. Other gastropod species, however, will be mentioned in this review, and, to orient the reader, **Table 1** shows their taxonomic context.

**Table 1:** Simplified taxonomy of the Gastropoda, showing the relative position and context of the species mentioned in the text.

Subclass	Order	Superfamily	Family	Genus
Patellogastropoda	Patellida		Patellidae	*Patella*
Vetigastropoda	Lepetellida		Fissurellidae	*Fissurella*
				*Megathura*
			Haliotidae	*Haliotis*
Caenogastropoda	Architaenioglossa		Ampullariidae	*Pomacea*
				*Marisa*
	Littorinimorpha		Littorinidae	*Littorina*
	Neogastropoda		Busyconidae	*Busycon*
			Fasciolariidae	*Fasciolaria*
			Muricidae	*Concholepas*
				*Rapana*
Heterobranchia	Hygrophila	Lymnaeoidea	Planorbidae	*Planorbarius*
				*Biomphalaria*
			Lymnaeidae	*Galba*
				*Lymnaea*
				*Radix*
			Physidae	*Physa*
	Eupulmonata	Helicoidea	Helicidae	*Cornu*
				*Helix*

Based on Bouchet et al. (3) and WoRMS (marinespecies.org).

Pioneering works on *Biomphalaria glabrata* concerning schistosomiasis had considerable influence in the second half of the 20th century (4–9). After that, most studies of gastropod immunity remained restricted to the Lymnaeoidea, in which impressive advancements have been made (10, 11), even though this may imply a strong taxonomic bias in our understanding of gastropod immunity.

In parallel, studies in those pioneering times extended to two caenogastropod species: the common periwinkle (*Littorina littorea*, Littorinidae) (e.g., 12) and the giant ramshorn snail (*Marisa cornuarietis*, Ampullariidae) (13). The work of Yousif et al. is of particular interest here because it was the first study of the immune cellular reactions of an apple snail. It dealt with the hemocyte reactions against larvae of *Angiostrongylus cantonensis* (Nematoda) infecting *M. cornuarietis.* Afterwards, ampullariid immunobiology reappeared as three studies of the defensive abilities of hemocytes of *Pomacea canaliculata* (14–16). These were the first research efforts concerning aspects of immunity in the widely diverse Caenogastropoda, which encompass about 60% of extant gastropod species (17, 18).

Notwithstanding, the development of gastropod “omics” studies in recent years, particularly the publication of the genomes of *B. glabrata*, *P. canaliculata*, and *M. cornuarietis* (19, 20), allows us to suppose that studies on these species will likely dominate the literature on gastropod immunobiology in the coming years.

## 3 The Gastropod Immune Systems From an Evo-Devo Perspective

We are aware of the fact that we are looking at the great diversity of gastropods through a keyhole (only a handful of species; **Table 1**), but we will dare to conjecture, on reasonable grounds, how the evolution of the gastropod immune system has been. Such an evo-devo attempt has not been made regarding gastropod immunity, and we think it is worth doing. With this aim, we will direct our attention to the reno-pericardial complex, because all potential hematopoietic sites have been proposed in derivatives of this complex (21).

In gastropods, the primordial reno-pericardial complex consists of a secondary body cavity (coelom) that grows and differentiates into the pericardium and the kidney/s. Next, from the dorso-posterior surface of the pericardium, the heart develops and fuses with the primary body cavity (hemocoel) (22). The pericardium remains connected with the kidney through a reno-pericardial canal, while the kidney chamber will become connected to the mantle cavity and the exterior, either directly (22, 23) or indirectly through a ureter (24). The latter does not derive from the reno-pericardial system but is an ingrowth of the pallial cavity (i.e., of ectodermal origin) (25, 26) and, therefore, it is a “secondary ureter” in the terminology of Ponder et al. (22). Ancestrally, the reno-pericardial ducts were simple ciliated canals (27), and the original tube-like structure, though with some expansions, can still be recognized in at least some Lymnaeoidea (5, 28). However, and particularly in the wide diversity of the Caenogastropoda, they became more complex as the distal portions of the duct were modified into sac-like kidneys (27), whose walls were lined by elaborate epithelial folds or papillae (22) or by deep epithelial crypts (as in the Ampullariidae; 29).

In our attempt to look at the gastropod immune system in an evo-devo perspective, we first hypothesize that the system has evolved in the mesenchymal connective tissue of the reno-pericardial complex. The components of this complex (i.e., the epithelial primordium and the mesenchyme accompanying it) seem to have evolved together in the Mollusca stem lineage and are ontogenetically and functionally related (27). And second, we also hypothesize that some hematopoietically committed progenitor cells have differentiated in the reno-pericardial complex, where they were able to proliferate and form (sometimes large) constitutive hemocyte aggregations at different, species-specific sites within the reno-pericardial territory.

We distinguish those aggregations as constitutive, because they are present in all healthy individuals of a species, as opposed to other, contingent aggregations that, in principle, can be formed in any organ but only as a response to intruders, and therefore, they are not always present in all individuals.

Some cells in the constitutive aggregations will remain as hematopoietically committed ones in adulthood, while some other may potentially differentiate into phagocytes, cells with lytic properties, etc. (whose features are not yet fully defined in gastropods). Also, there can be in-and-out cell migrations from the constitutive hemocyte aggregations, so that there would be some equilibrium between them and the circulating blood, and they may work as hemocyte reservoirs (section **4.1.3.2**). In this way, these hemocyte aggregations, if retaining hematopoietically committed progenitor cells, may also contribute to sustain the circulating hemocyte count within physiological ranges or may export hematopoietically committed progenitor cells to distant sites in the hemocoel and connective tissue, where they may differentiate in response to intruders. In this way, this evo-devo approach may reconcile the views of those authors proposing the occurrence of localized hematopoiesis in some species (e.g., 30) or spread in others (e.g., 31). Also, there may be some species, such as *P. canaliculata*, where both localized (29) and spread hematopoiesis (32) may occur.

This evo-devo hypothesis, intended to understand molluscs and particularly gastropods, will be central in this review, and is in line with Hartenstein’s (33) broader views on the Metazoa, but which paid little attention to Lophotrochozoa, and to Mollusca in particular.

## 4 The Ampullariid Immune System From a Morpho-Functional Perspective

Seemingly different arrangements of the immune system occur in all metazoan phyla and serve diverse functions besides the resistance to intruders. In gastropods, there is evidence of hemocytes participating in shell growth, tissue remodeling and repair, and the movement of metabolites and nutrients (10, 33, 34). However, we here examine only two central morpho-functional aspects of the immune system of Ampullariidae. First, we consider *what* constitutes the defense system, including the processes of eliminating or walling off intruders. Second, we discuss *where* and *how* the defensive cells (the hemocytes) originate and proliferate, either to maintain nearly constant numbers in the blood or to overcome infections.

### 4.1 The Defense System

#### 4.1.1 Organs and Cells

A first defense barrier in metazoans is the integument, a physical barrier that in gastropods is reinforced by the shell and by mucus secretion, which is an energetically costly but considerably effective defense against intruders (10, 35). Most gastropods are aquatic, and their gills are a specialized part of the integument continuously swept by the pallial water current (23) that may also oppose the penetration of intruders. In *P. canaliculata*, the gill is loaded by intraepithelial granulocytes (36) that may also have a role in opposing intruders.

Beyond the integument, the defense system consists of hemocytes (either resident or circulating) and of humoral factors, which are produced either by the hemocytes themselves or by associated epithelial or connective tissue cells (35). Hemocytes eliminate or wall off intruders through different processes that overlap to some extent. For example, elimination may occur through phagocytosis followed by intracellular lysis. Extracellular lysis may include the production of lytic enzymes or reactive oxygen or nitrogen species, among others.

Phagocytosis, originally described by Metchnikoff (37) in a variety of organisms, is certainly considered a phylogenetically ancient process because it occurs in many protists and all metazoans studied so far. Phagocytosis is usually followed by intracellular lysis of the engulfed intruder (38). Extracellular lysis of intruders can occur after degranulation of hemocytes, which release lytic enzymes that may act on surrounding intruders or even systemically, as suggested by Ottaviani (39). Degranulation can occur through simple or even compound exocytosis (40, 41). Also, the release of reactive oxygen species by activated leucocytes may cause the external lysis of intruders (42). A more recently discovered mechanism involving cell death is the extrusion of an “extracellular trap” of DNA and lytic enzymes (=ETosis) in which intruders are captured and lysed (43–46). It seems widespread among Metazoa and thus is considered a potentially ancient process. However, contrasting results have been reported in gastropods: while ETosis by hemocytes of three stylommatophoran gastropods (genera *Achatina*, *Arion*, and *Limax*) was effective against metastrongyloid larvae *in vitro*, hemocytes from three lymnaeoid species (genera *Lymnaea*, *Planorbarius*, and *Radix*) failed to generate ET-like fibers in response to a wide range of *in vitro* stimulants (47, 48).

#### 4.1.2 Hemocyte “Populations” and “Types” in *P. canaliculata*

The morphological variations of gastropod hemocytes defy any succinct description and classification (21, 35). Disagreements may be due to some real differences (e.g., species or developmental differences) but may also result from the different criteria and nomenclatures adopted by researchers. The main difficulties result from the lack of biological markers to specifically diferentiate cell lineages or states of maturation (32). Years ago, we proposed a synonymy of the names in use for architaenioglossan species (41), and we used the designations of Cheng (49) for *B. glabrata*. However, our proposition was not followed.

Besides the confusion caused by the multiple and arbitrary naming systems, a risk that anyone developing a new classification system must avoid is the temptation to forcibly fit the data into preconceived molds. And one such mold may be the mere existence of hemocyte “types” rather than hemocyte “populations”, whose morpho-functional traits vary around some central, more frequent ones. In that context, a hemocyte type should get represented by the cells that express those more frequent traits.

**Figure 1:**
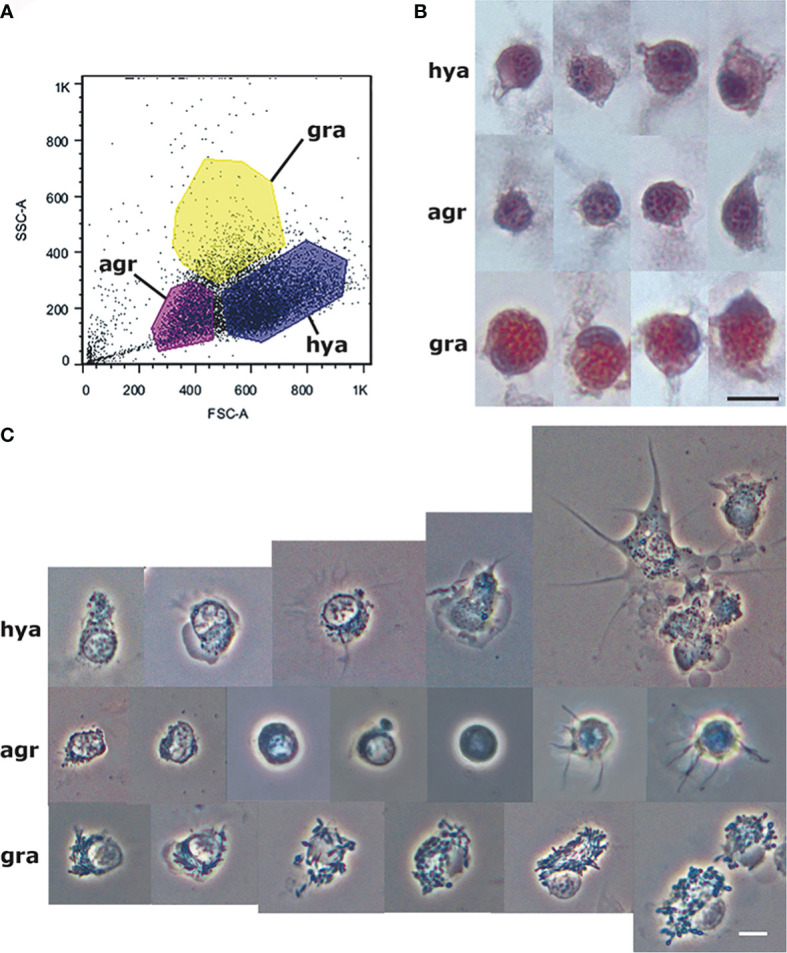
Circulating hemocyte populations of *P. canaliculata* and their separation by flow cytometry. **(A)** Flow cytometry (dot plot of size vs. complexity-granularity) of a representative blood sample indicating the sorted areas where either agranulocytes, granulocytes and hyalinocytes were predominant. **(B)** Examples of hyalinocytes, agranulocytes and granulocytes in hemolymph smears (hematoxylin-eosin stain). **(C)** Examples of living hyalinocytes, agranulocytes and granulocytes attached onto a glass slide (phase contrast). GR, agranulocytes; GRA, granulocytes; HYA, hyalinocytes. Scale bars represent 5 μm. From (41).

Then, we wanted to know if, in flow cytometry scatterplots, we could distinguish different populations of circulating hemocytes, i.e., groups of events differentiated by their central (more frequent) values, by merely separating the cell events for their size and complexity/granularity (i.e., forward and side scatter, respectively). For that purpose, blood samples were drawn with a sterile syringe soaked with an antiaggregant solution directly from the heart ventricle (41). The mean hemocyte concentration in the blood obtained under those conditions was ~2900 cells/µL, and the blood samples were sterile, as suggested by no cultivable bacterial growth found in subsequent cultures (15). According to our expectations, some different populations emerged in plots (**Figure 1**). The subsequent study of fixed and sorted live cells showed that the cell populations could be allotted to the hemocyte “types” Cheng referred to for *B. glabrata* (49), i.e., “hyalinocytes,” “granulocytes,” and “agranulocytes”. That was also in keeping (though under different names) with the observations of George and Ferguson (50) in three neogastropod species (*Busycon carica*, *Busycon canaliculatum*, and *Fasciolaria tulipa*).

**Figure 2:**
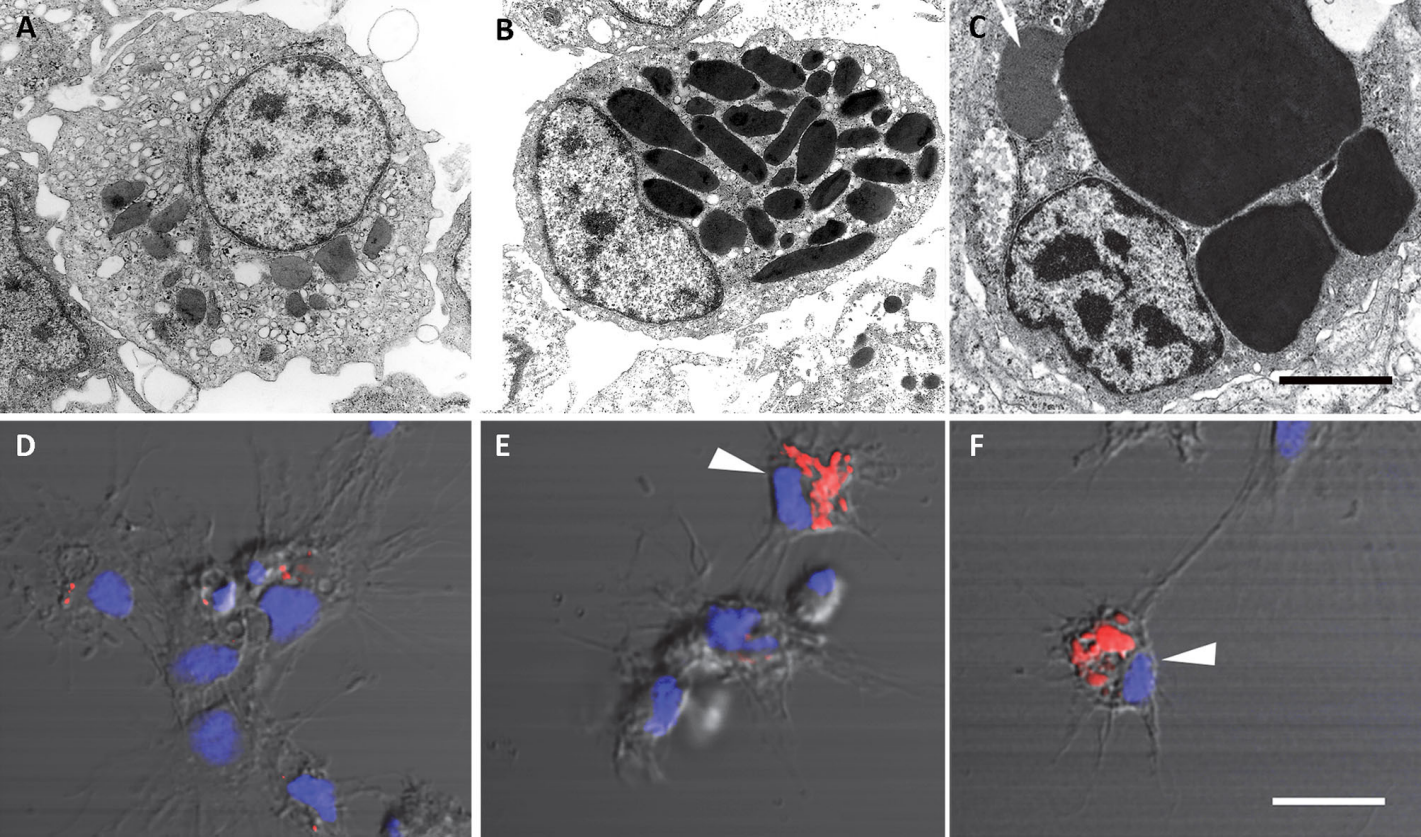
Circulating hemocytes of *P. canaliculata*. Panels **(A–C)**, TEM; scale bar represents 1 μm. **(A)** Hyalinocyte with an eccentric nucleus, numerous SER vesicles, mitochondria and L granules; a few profiles of the RER are also seen. **(B)** Granulocyte, showing a displaced, bean-shaped nucleus and numerous R granules; some SER vesicles are also seen. **(C)** Granulocyte in a preparation exposed to *E. coli* cells showing extensive R granule fusion and a single L granule (arrow). Panels **(D–F)**, laser confocal microscopy, LysoTracker Red-Hoechst 33258 staining; scale bar represents 10 μm. **(D)** A group of spreading hyalinocytes; small acidic L granules (red) are seen in some of them. **(E)** A spreading granulocyte (arrowhead) showing numerous rod-shaped, acidic granules; also, there is a group of spreading and round hyalinocytes, which are essentially devoid of acidic granules. **(F)** Another spreading granulocyte (arrowhead) showing large and merging acidic granules; also, there are two spreading hyalinocytes with no acidic granules. Micrographs from (41).

**Figure 3:**
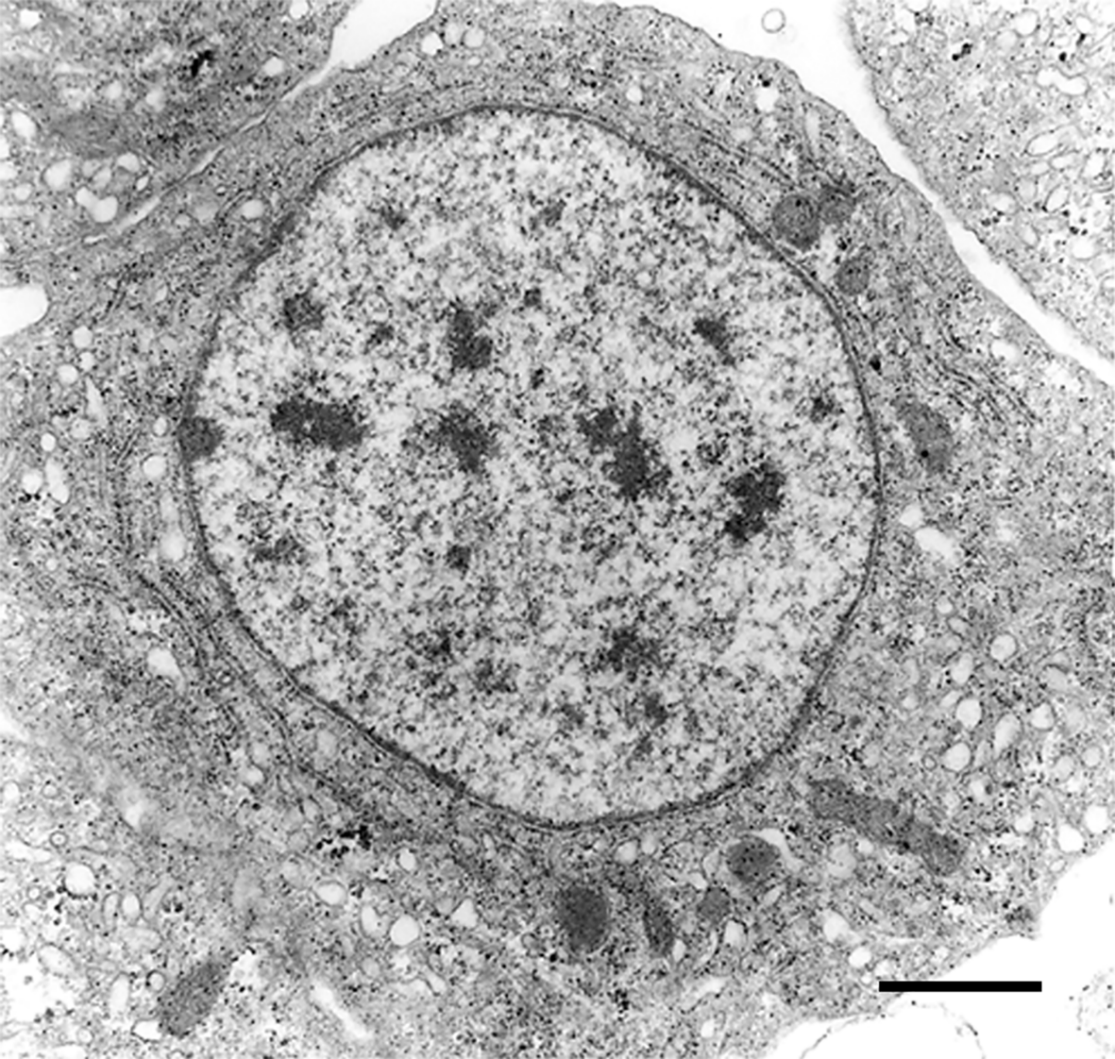
A circulating agranulocyte of *P. canaliculata* with a round central nucleus, SER vesicles and RER cisternae, as well as some cytoplasmic granules (under TEM). Its ultrastructural features are compatible with the quiescent cells that have been described in the circulation (32). Scale bars represent 1 μm. Micrograph from (41).

The recovery of these populations after flow cytometry and cell sorting allowed the observation (41) not only of their tinctorial affinities (both in living and fixed material) but also the behavior of living cells under phase-contrast microscopy (**Figures 1A, B**). Hyalinocytes were the most actively moving cells and were frequently emitting large lamellipodia and filopodia (**Figure 1B**). The nuclei were either round or bean-shaped (**Figures 1A, B**). Cytoplasmic microgranules were not clearly distinguished in the hematoxylin–eosin preparations but were visible under phase-contrast microscopy and were likely the mitochondria and the small, lysosome-like granules (L-granules; 41) that were disclosed under transmission electron microscopy (TEM) (**Figures 2A–C**). The L-granules were also clearly seen when observed under laser confocal microscopy after staining with LysoTracker Red, which allows detecting acidic compartments (**Figures 2D–F**). Granulocytes, in turn, showed a low nucleus/cytoplasm ratio and numerous large eosinophilic granules (**Figure 1A**). These granules were also visible as large dark spots under phase-contrast microscopy (**Figure 1B**), and as granules of high electron density under TEM [called R granules, (41)]. These granules were also deeply stained with LysoTracker Red (**Figure 2E**). Agranulocytes showed a large nucleus/cytoplasm ratio and no apparent cytoplasmic granules in fixed-stained material; however, occasional filopodia could be seen (**Figures 1A, B**). Under TEM, agranulocytes showed round nuclei and scant cytoplasm with few mitochondria and cytoplasmic L granules (**Figure 3**). The agranulocyte population is worthy of attention because it likely contains both dividing and quiescent cells, which eventually may act as progenitor cells, either in the circulation or in the hematopoietic tissues/organs (32).

**Figure 4:**
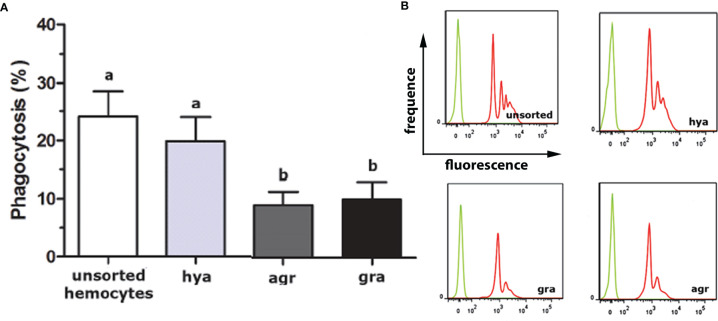
*In vitro* phagocytosis of fluorescent beads by unsorted hemocytes of *P. canaliculata* and by sorted circulating hyalinocytes, agranulocytes and granulocytes. **(A)** Phagocytosis index of unsorted and sorted circulating hemocytes (means ± SEM; N = 6; different letters indicate statistically significant differences, one-way ANOVA, Tukey test). **(B)** Histograms showing the distribution of phagocytic (red) and non-phagocytic (green) hemocytes. Fluorescence peaks correspond to hemocyte subpopulations with different numbers of phagocytized fluorescent beads. GR, agranulocytes; GRA, granulocytes; HYA, hyalinocytes. From (41).

The proportions of the three hemocyte populations in the circulation, calculated on blood samples fixed and stained on glass slides, were: hyalinocytes, 63%; agranulocytes, 28%; and granulocytes, 9% (41). The phagocytic index, i.e., the percent of phagocytizing cells, was significantly higher in hyalinocytes (**Figure 4A**) after fluorescent bead exposure of sorted populations of circulating hemocytes. The different populations were phagocytizing different numbers of beads, as indicated by distinct fluorescence peaks (**Figure 4B** **).**

There was a previous paper on circulating *P. canaliculata* hemocytes (16) that provided an alternative view. That study differed from ours in the way they collected blood (and hence in the quality of the blood samples) and in the procedures to which the cells were submitted afterward. Blood withdrawal in (16) was performed by applying “a gentle but continuative pressure” on the operculum until a blood quota was released and which could be collected with a pipette. A similar procedure has been used to sample blood from smaller, non-operculate snails (51–53) in which the body withdrawal into the shell is accompanied by the expulsion of blood through a “hemal pore” (54, 55). This method may be the only one convenient for such small snails and, for ampullariids, it has the important experimental advantage of possibly obtaining repeated blood samples from the same animal. However, the quality of blood obtained in such conditions is not as good as that from the ventricle, because it may be diluted with urine and contaminated with dust and microorganisms from the snail’s shell and operculum. In the case of *P. canaliculata*, dilution was evidenced by the much lower hemocyte counts obtained with this method, as compared with blood obtained from the ventricle (~1100 *vs.* ~2900 cells/µL), and contamination was shown by the high background of flow-cytometry events, which was similar, or even exceeded that of the hemocytes (16; their Figure 1). Also, hemocytes showed phaocytosis profiles under TEM (16; their Figures 8C, D), while blood obtained from the heart is sterile (15). In their conditions, the authors could separate two circulating hemocyte populations (identified as Group I, of smaller hemocytes, and Group II of larger ones). Other potentially useful subgroupings (“small” and “intermediate” for Group 1, and “agranular basophilic”, “agranular acidophilic” and “granular” for Group 2) were made on fixed and stained hemocytes (the size limits to separate the “small” and “intermediate” cells within Group I were not stated). Even after the more complete study was published (41), they continued to refer only to their paper, until recently (56), when ours was mentioned just for the buffer we used.

The latter paper (56), however, introduces an image-based method (named Image3C, for Image-Cytometry Cell Classification), which is able to distinguish populations in a cell suspension without the aid of species-specific reagents, such as antibodies. Therefore, the method will be suitable to overcome many difficulties that have been presented in the study of *P. canaliculata* as well as other important species in which basic tools for cell studies are scanty. Image3C combines image-based flow cytometry with an unbiased, high-throughput cell clustering pipeline followed by convolutional neural network (CNN) processing (56). CNN is an artificial intelligence method that has the capacity to analyze images and recognize patterns within them. They ran circulating hemocytes of *P. canaliculata* through Image3C and found nine different hemocyte clusters. The authors attempted to correlate the new findings with those obtained by them with other methods (16), but did not attempt any correlation with the more informative study by Cueto et al. (41). Their method, however, may represent an important breakthrough in the study of ampullariid hemocytes, which may lead to a deeper understanding of these cells, and may allow the recovery of different hemocyte subpopulations and of raising antibodies that would enable to recognize the different subpopulations immunologically.

A significant contribution that will be referred later in this review, is the first description of the proteome of *P. canaliculata* hemocytes (57).

#### 4.1.3 Internal Organ Barriers, Hemocyte Reservoirs, and Nodules

##### 4.1.3.1 Constitutive Hemocyte Aggregations: The Renal Hemocyte Islets

**Figure 5:**
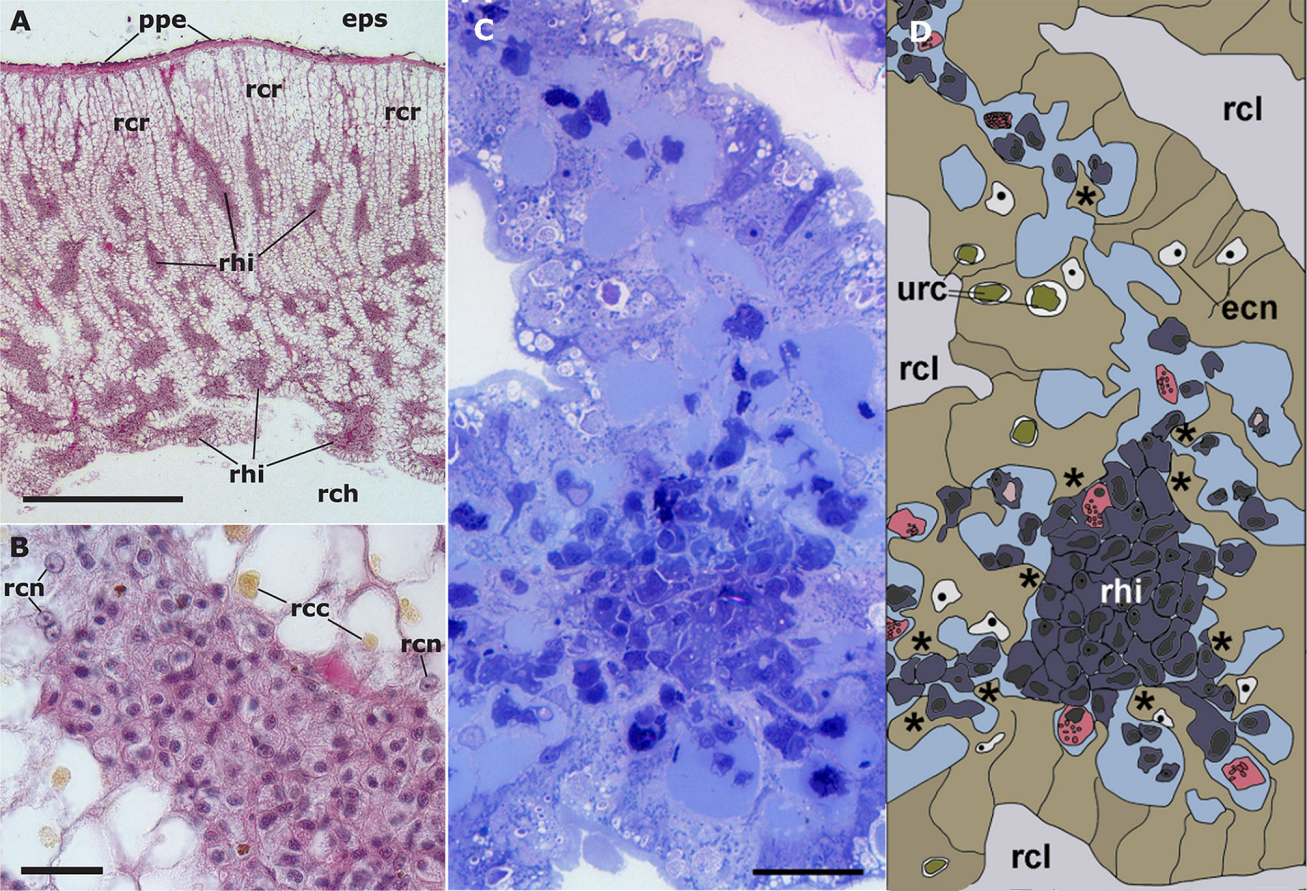
Constitutive hemocyte islets (panels **(A, B)**, paraffin and hematoxylin-eosin; panel **(C)**, Spurr resin and toluidine blue). **(A)** Section of the dorsal renal epithelium, perpendicular to the outer surface of the organ. The pigmented outer mantle epithelium separates the kidney from the extrapallial space. Renal hemocyte islets are seen as elongated basophilic masses between the cortical renal crypts, while they appear transversally sectioned in the region overlying the renal chamber. **(B)** Section of a hemocyte islet in the space between two epithelial crypts, at higher magnification. **(C)** Section through a medium-sized hemocyte islet which is retained in place by basal extensions of the renal epithelial cells. **(D)** Diagram of the same section shown in **(C)**, highlighting a hemocyte islet, together with some smaller aggregations; the entire set is retained in place by cytoplasmic extensions (asterisks) of renal epithelial cells (light brown); the space for the flowing blood plasma is shown in pale blue. Hyalinocytes are shown in dark blue while granulocytes are shown in pink. Scale bar represents 20 µm. ECN, epithelial cell nuclei; EPS, extrapallial space; PPE, pigmented pallial epithelium; RCC, renal cell concretion; RCH, renal chamber; RCL, renal crypt lumen; RCN, renal cell nucleus; RHI, renal hemocyte islet; URC, urinary concretions. From (29, 41).

**Figure 6:**
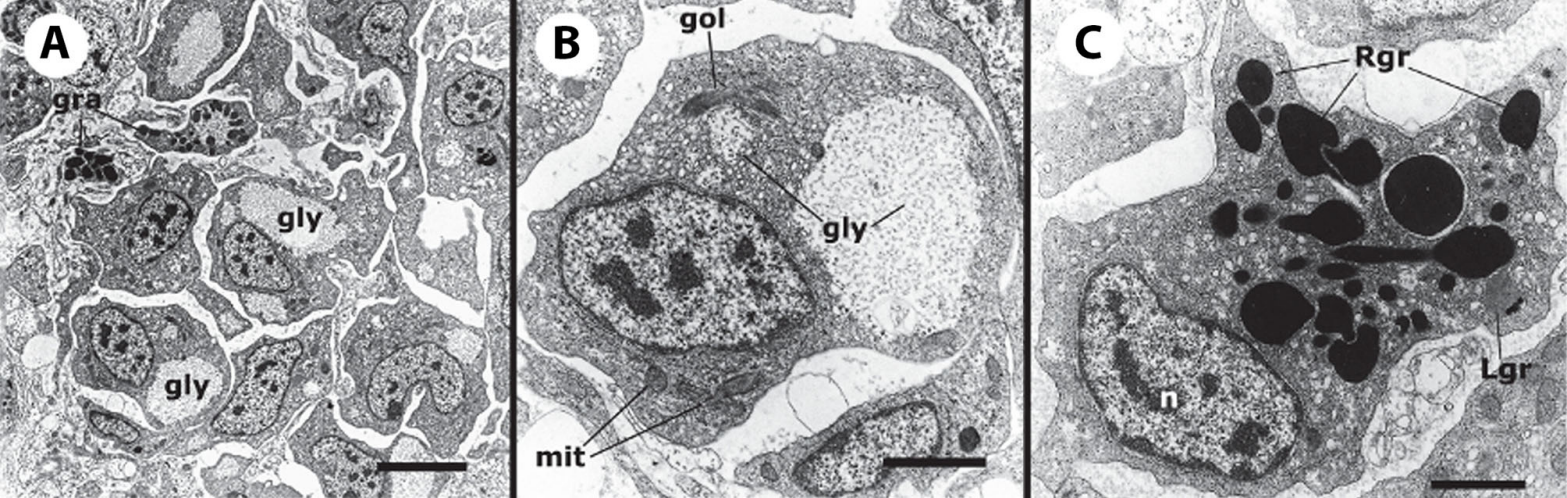
Constitutive hemocyte islets (panels A-C, under TEM). **(A)** Numerous hyalinocytes and two tangential sections of granulocytes at the core of a renal islet. Non-membrane bound areas with presumptive glycogen clumps appear in most cells and are larger than those in circulating hemocytes. **(B)** Detail of a hyalinocyte from the preceding panel, showing a Golgi stack, areas of presumptive glycogen clumps, SER vesicles and mitochondria. **(C)** A granulocyte in a renal islet, showing SER vesicles, some RER profiles and numerous R granules of different sizes (some of them appear merging); a single L granule is also seen. Microgranular material and membrane remnants are found in the intercellular space.AGR, agranulocyte; GLY, presumptive glycogen granules; GOL, Golgi stack; GRA, granulocyte; HYA, hyalinocyte; LGR, L granule; RGR, R granule. Scale bars in **(A, B)** panels represent 5 μm, while those in other panels represent 1 μm. From (41).

The dorsal renal epithelium is the largest portion of the kidney and shows conspicuous hemocyte islets in the hemocoelic spaces between the epithelial crypts (41). A field view of the dorsal renal epithelium of *P. canaliculata* is shown in **Figure 5A**. These hemocyte aggregates are a constitutive part of the kidney of healthy individuals and are composed of agranular cells (hyalinocytes, agranulocytes) and just a few granulocytes (**Figures 5B–D**). The islets are interposed in the blood drainage of this major part of the kidney (29) where they are loosely arranged and anchored by basal extensions of the renal epithelial cells. These epithelial extensions show no cellular junctions, which may facilitate the in-and-out hemocyte migrations, while the entire islet remains in place because of the network of basal epithelial extensions (**Figures 5C, D**) (29). Also, some of the circulating progenitor cells (32) may be caught in that network. Many hyalinocytes in the islets, but not granulocytes, contain non-membrane-bound cytoplasmic areas that are likely glycogen deposits and may have a role in storing this energetically valuable substance (**Figures 6A–C**).

Similar hemocyte aggregates have been observed in other gastropods since the initial observations of Perrier in *L. littorea* (58), and though under different organ names, there were always associated with reno-pericardial derivatives (59–61).

**Figure 7:**
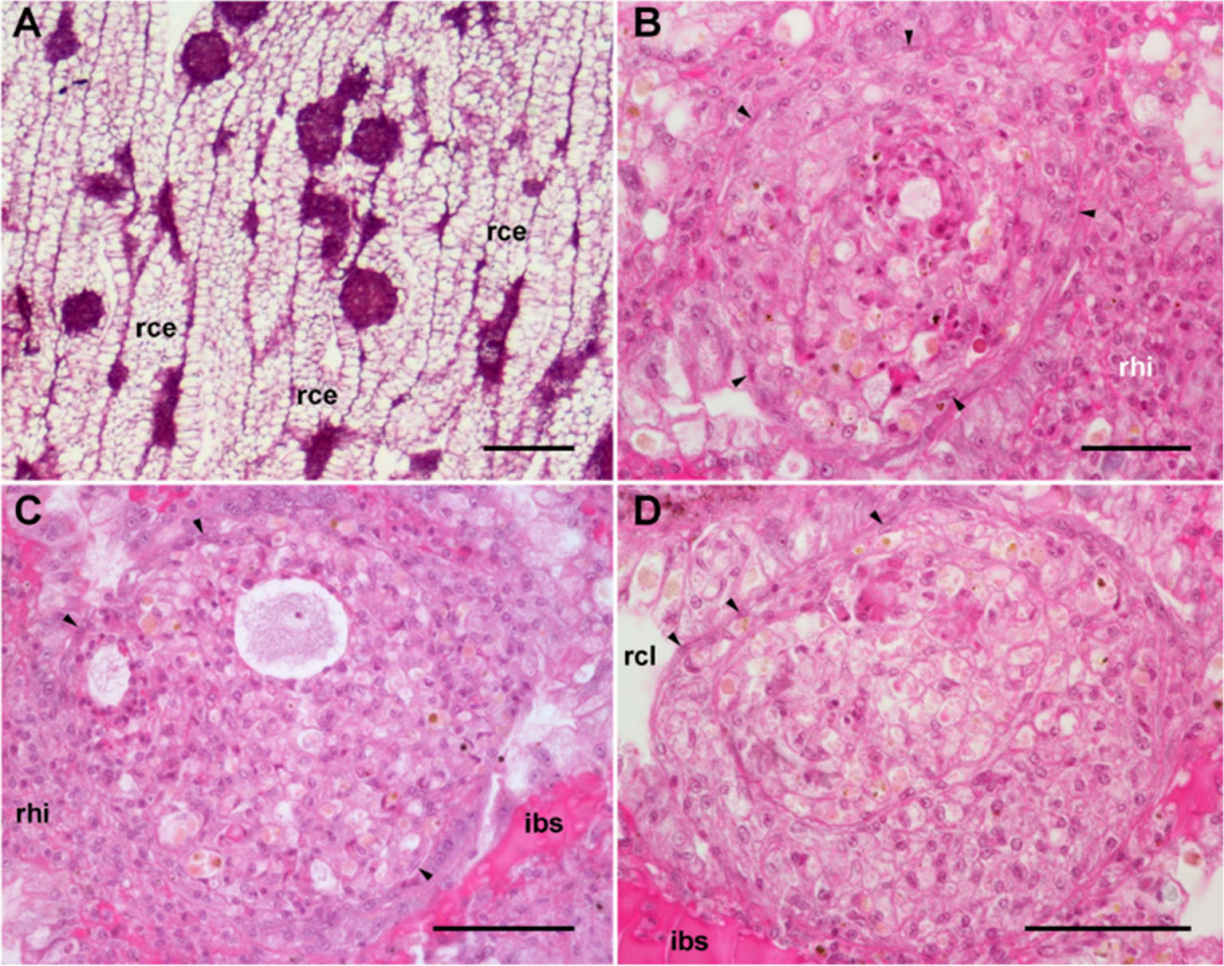
Nodular hemocyte reactions after an immune challenge (hematoxylin-eosin stain). **(A)** Field view of the dorsal kidney epithelium showing enlarged islets and nodules 96 h after yeast inoculation. **(B, C)** Detail of hemocyte nodules in another treated snail showing acellular cavities, apoptotic bodies, lipofuscin-like deposits, some granulocytes, and a delimiting band of flattened cells (black arrowheads). The nodule is pushing against the cavity of a crypt in **(B)**, while is in contact with a blood sinus in **(C)**. **(D)** A nodule showing a core and a cortex; the core appears to be in a more advanced stage of regression; bands of flattened cells separate the core from the cortex, and the cortex from the surrounding tissues and a blood sinus. Scale bars represent: **(A)** 200 µm; **(B–D)** 50 µm. IBS, intercryptal blood sinus; RCE, renal cryptal epithelium; RCL, renal cryptal lumen; RHI, renal hemocyte islet. From (29, 41).

The area of the constitutive hemocyte aggregations in the kidney significantly increases after an immune challenge (inoculation of yeasts, *Saccharomyces cerevisiae*) to *P. canaliculata* (29), that may indicate either attraction of hemocytes to these aggregations or the generation of new cells within the islets, i.e., hematopoiesis, as it will be reviewed in section **4.2**. Also, hemocyte nodules are formed as outgrowths of these islets **(** **Figure 7** **)**, sometimes dislodging the renal epithelium, so that the nodule becomes in direct contact with the cryptal lumina and thus with the renal cavity. In *P. canaliculata*, we have substantiated the existence of internal “immune barriers”, referring to those organs that are able to prevent the dissemination of intruders, either because of its position in the circulation, or because their intricate and stagnant microcirculation, which would increase the probability of hemocyte contact with the intruders (29). These organ barriers are here called “internal” to distinguish them from the large “external” organ barrier formed by the shell and the integument (section **4.1.1**).

In *P. canaliculata*, the kidney is an immune barrier with constitutive hemocyte aggregations, which makes it an analog of lymph nodes in mammals. Indeed, these nodes are interposed in the lymph flow, which stagnates in their sinuses as the renal hemocyte islets are interposed in the blood flow of the ampullariids. Another analogy is that the lymph of mammals and the blood of ampullariids both find a locally high concentration of immunocompetent cells when confronting the barrier, which makes them more effective.

##### 4.1.3.2 Constitutive Hemocyte Aggregations as Hemocyte Reservoirs

It has been reported (62) that repeated bleeding (2 mL each) within two consecutive days did not significantly affect the circulating hemocyte concentration in *P. canaliculata*, which would imply that capacious hemocyte reservoirs should exist in this snail, that would be able to release hemocytes to the circulation, thus contributing to homeostasis.

Constitutive hemocyte islets found in the kidney (**Figure 5**) (41) are candidates for this function in *P. canaliculata* because they are major hemocyte aggregations that could contain sufficient cells to significantly affect the circulating concentration after hemocyte release. These islets are contained within the hemocoelic draining spaces of the kidney, where they are retained by a meshwork of basal extensions of the renal crypt epithelium, but with no intervening intercellular junctions (**Figure 5**) (29). Also, some of the circulating progenitor cells (32) may be caught in that network and would be the basis of the role of these islets as hematopoietic sites (see section **4.2.3.2**). Likewise, *in vitro* observations of hemocyte aggregates showed a dynamic exchange of cells by diapedesis, between the aggregate and the immediate environment (see section **4.1.3.3**). By diapedesis, the hemocytes may also leave the islet traversing the mesh of epithelial projections that hold the hemocyte islet in place. Afterward, the hemocytes can be carried away with blood drained from the kidney, which is collected by the efferent renal vein and goes directly to the heart auricle and hence to the general circulation. It would be crucial, however, to determine the release of marked cells from the islets in response to peripheral depletion of hemocytes.

Alternatively, it has been proposed (62) that the aortic ampulla would serve both as a hemocyte reservoir and as a site of hemocyte maturation (the ampulla is an extensible outgrowth of the pericardial portion of the anterior aorta, and this small organ is a synapomorphy of the Ampullariidae). We think that the proposition of the ampulla as a reservoir was based on a misinterpretation of the storage cells’ nodules occurring in the ampullary walls (63). In fact, the latter study combined light and electron microscopies, subcellular fractionation, and biochemical determinations, and could indeed show that the cytoplasm of these large cells is loaded with urate crystalloids. On the basis of this study one may reinterpret the light microscopy observations of (62) as follows: (a) the “white spots” shown in their Figure 3A would correspond to the nodular urate tissue described in (63); (b) the “compartmentalized lobules” would correspond to the large storage cells whose cytoplasm is extracted in light microscopy preparations (63); and (c) the stocked “hemocytes” would correspond indeed to the nuclei of these storage cells, which may still retain a cytoplasmic rim. These reinterpretations were further supported by a recent TEM study of the same group that confirmed the occurrence of storage cells and crystalloids, but no hemocytes, in the walls of the aortic ampulla (64).

Regarding the proposed role of the aortic ampulla as a site of hemocyte maturation (62), we think it was proposed not as a formal hypothesis but as a mere possibility, and it has no empirical support. Curiously (and wrongly), the same idea has been attributed to some of us in a rather recent paper (65), but it has not been mentioned in another recent study by the same group (64) and so it was probably abandoned.

##### 4.1.3.3 Contingent Hemocyte Aggregations: Nodules (*In Vivo* and *In Vitro*)

In principle, however, contingent hemocyte aggregations can occur anywhere in the body, but are certainly facilitated in the internal immune barriers, such as the lung in *P. canaliculata*, an organ that lacks constitutive hemocyte aggregations, but where large hemocyte nodules are also formed in response to yeast injection (29). Indeed, nodule formation in the lung relies on the stagnant circulation of the respiratory lamina, an extensive network of blood sinuses serving gas exchange (29). Therefore, nodulation in the lung follows a somewhat different pathway, because small hemocyte aggregations form within the large vessels supplying the organ, and these aggregations are later dislodged and clogged in the narrower sinuses of the respiratory lamina, where they may grow by the local generation of hemocytes or by the attraction of circulating or wandering ones (29).

**Figure 8:**
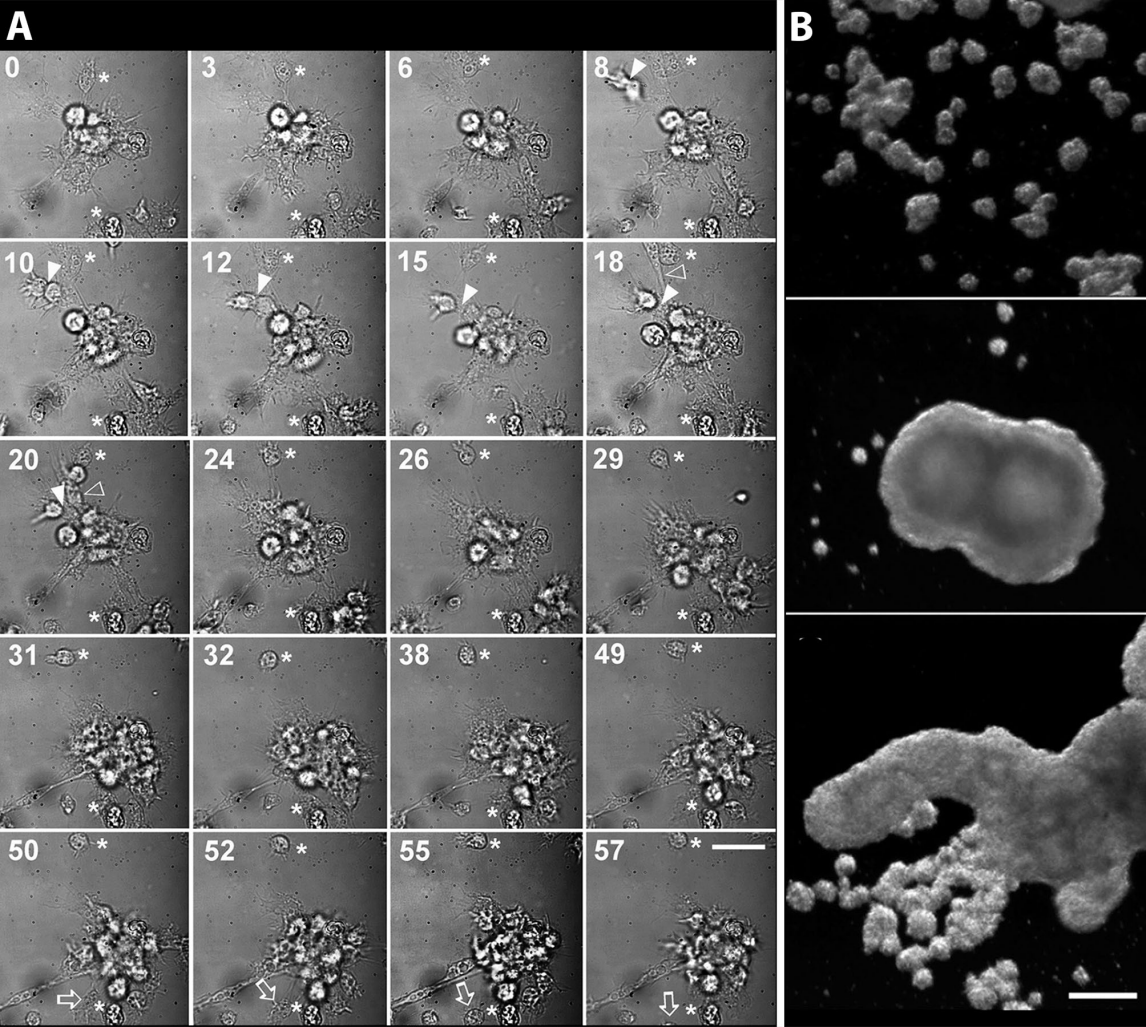
Circulating hemocytes of *P. canaliculata* in culture. **(A)** Dynamics of an early aggregate formation by time-lapse video-microscopy. Time elapsed after seeding (in minutes) is shown in the upper-left corner of each selected frame. Arrowheads indicate two cells together which are approximating the larger aggregate and finally merge with it (8–20 min). Open arrowheads show an elongated cell which rapidly joins the aggregate (18–20 min). Open arrows point to a cell which detaches from the aggregate and leaves the observation field (50–55 min). Also, a smaller cell aggregate appears in the lower right corner approximating the larger aggregate and finally joining it (18–31 min). Asterisks indicate two cells which appear attached to the substrate and did not move during all the period of observation (0–57 min). Scale bar represents 20 µm. **(B)** Hemocyte aggregates formed in culture and merging into large floating masses (phase contrast, micrographs taken 96 h after seeding). Scale bar represents 200 µm (all panels). From (15).

Cueto et al. (15) developed a method for the primary culture of circulating hemocytes of *P. canaliculata*, which may be interesting to study nodulation. Hemocytes in those conditions showed the evolutionarily conserved behaviors of attachment, pseudopodia emission, and phagocytosis. Real-time, time-lapse videomicroscopy showed the dynamics of the early hemocyte aggregations, with cells both entering and leaving the nodules (**Figure 8A**). Besides, these cells showed a marked tendency to form large nodular aggregates that may later merge in masses that could be visible with the naked eye (**Figure 8B**).

**Figure 9:**
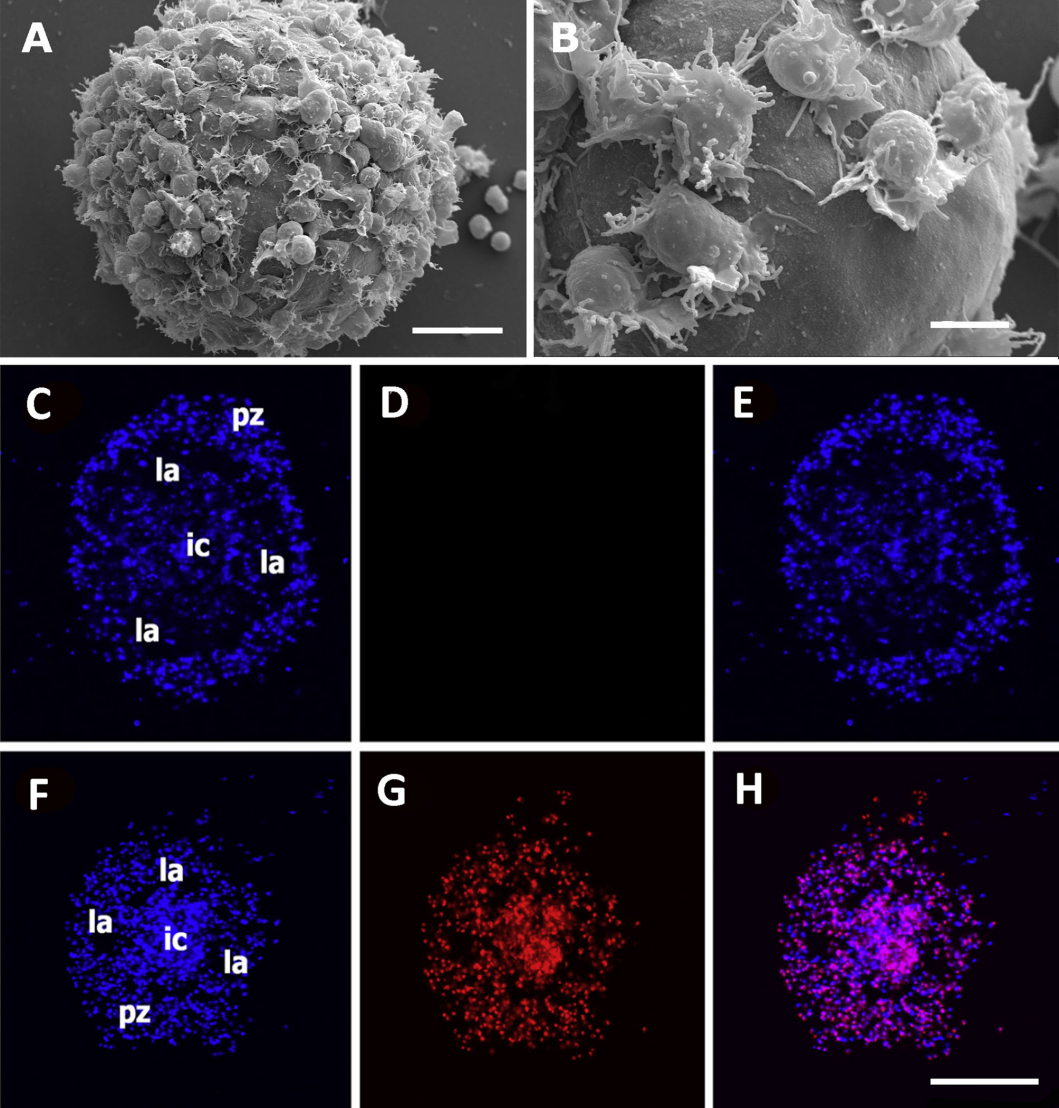
Hemocyte aggregates *in vitro*. **(A)** Large nodule mostly covered by filopodia/lamellipodia emitting cells, 96 h after seeding; scanning electron microscopy; scale bar represents 20 µm. **(B)** Detail of the smooth external aspect of a nodule, with some filopodia/lamellipodia emitting cells on it, 96 h after seeding; panels **(A, B)** are scanning electron microscopy micrographies; scale bar represents 10 µm. Panels **(C–H)** Floating hemocyte aggregates in culture, 72 h after seeding (DAPI and propidium iodide staining; laser confocal microscopy). **(C)** Hemocyte nodule grown *in vitro* showing the peripheral cell zone, the intermediate lacunar zone, and the inner core (DAPI emission, blue). **(D)** Same hemocyte aggregate lacking propidium iodide staining (red emission), indicating that all cells were viable. **(E)** Merging of micrographs **(C, D)**. **(F)**. Another aggregate showing the same basic organization (DAPI emission, blue). **(G)** Same aggregate showing abundant cells marked with propidium iodide (red emission), indicating that most cells in this aggregate were not viable. **(H)** Merging of micrographs **(F, G)**. Scale bar for panels **(C–H)** represents 100 µm. IC, inner core; LA, lacuna; PZ, peripheral zone. From (15).

Both the large single nodules and the merging ones detach from the bottom surface and float freely in the medium, while migrating cells emitting lamellipodia and filipodia occur near their surface (**Figures 9A, B**). The nodules may show a central compact zone, an intermediate zone with large extracellular lacunae and an outer zone of flattened cells (15). Dual DAPI/propidium iodide staining revealed the coexistence of nodules with varying proportions of viable and non-viable cells (**Figures 9C–H**). Their formation in axenic culture parallels the *in vivo* generation of hemocyte nodules in response to immune challenges (**Figure 7**) (29), although an obvious difference is that nodule formation in axenic cultures could only be elicited by some kind of contact factor.

##### 4.1.3.4 Nodulation in Other Metazoan Taxa and Other Hemocyte Aggregations

Nodular hemocyte formations are known to occur in the major clades of Protostomia (e.g., 66–69) and Deuterostomia (e.g., 70). A particular case among the latter are the nodules formed in the zebrafish larvae (*Danio rerio*, Teleostei, Cyprinidae) in response to *Mycobacterium marinum* inoculation, because these nodules (usually called granulomas or tubercles in the context of tuberculosis studies) have received a particular attention as a model of mycobacterial pathogenesis (71–75). Since the zebrafish larvae lack adaptive immune system early in ontogenesis, it is thought that the initiation of nodulation can occur without any contribution of the adaptive immune system (72). The same can be said of nodules induced by different stimuli in *P. canaliculata* and other invertebrates, where an adaptive immune system would never develop. Also, Ramakrishnan (76) elaborated on the idea that though the tuberculous granuloma was traditionally thought as a protective structure committed to wall-off and destroy the tuberculous bacilli, they may also provide a suitable environment for the multiplication of this intracellular parasite and also, that the in-and-out migration of macrophages from the nodule would result in the dissemination of the infection, rather than on its contention. Similar in-and-out hemocyte migration has been observed during the early phases of nodule formation in *P. canaliculata* (15) (see section **4.1.3.3**). Therefore, Ramakrishnan’s ideas should be considered while exploring the nodular reactions to intruders in gastropods and other invertebrates.

Besides nodule formation, diffuse hemocyte infiltration of the connective tissue has been reported in *M. cornuarietis* in response to *A. cantonensis* infection (13), but this was not frequent in *P. canaliculata*, at least in response to yeasts (29). The single report of the cellular reactions of *M. cornuarietis* to an infection indicated a first phase of loose hemocyte aggregations around the larvae of *A. cantonensis* (first 24 h after exposure) that was followed 10-40 days later by massive granulocyte aggregations within large hemocoelic spaces and the formation of nodules by non-granular hemocytes in the connective tissue. A follow-up of these observations and a closer comparison with the cellular reactions in *P. canaliculata* is therefore needed.

#### 4.1.4 Lectins, Complement, and Other Soluble Immune Factors

Lectins, first known in plants (77), are currently known in all kind of organisms and serve different functions. They were first appreciated for their cell agglutinating ability, which attracted medical interest for the diagnosis of blood groups in humans (78). In molluscs, they serve innate immune defenses, based mainly on their ability to specifically recognize hydrocarbon moieties on antigens expressed on the surface of bacteria, fungi or metazoan parasites (79). In gastropods, they may circulate in the blood (e.g., 80) and participate in defensive reactions such as cytolysis or phagocytosis of intruding cells and activation of the complement system *via* specific pathways (81). They have been well characterized in some gastropods, notably in *B. glabrata* (11).

In the Ampullariidae, medical interest was also the initial driver of scientific attention, because erythrocyte agglutinating activity was first reported in “expressed tissues” (*sic*) of *Pomacea bridgesii* (82), but their discovery in extracts of the albumen gland of the congeneric *P. paludosa* and *P. urceus* (83, 84) led research in a different direction, because those lectins could pass to the perivitelline egg components, and may play a defensive role for embryos that these species incubate above water level, and are thus exposed to a variety of biotic and abiotic threats. The defensive role of perivitellins in *Pomacea* eggs was first recognized as part of a multifunctional protein complex that defends the embryos during incubation (e.g., 85–89). One of these proteins, named PcPV2 [for *P. canaliculata* perivitellin 2 (90)], is a potent neurotoxin combining lectin and pore-forming chains that would participate in deterring predators. Also interesting, a lectin found in *P. canaliculata* that may be exclusive of Caenogastropoda, combines two immunoglobulin domains and the scavenger receptor Cys-Rich (SRCR) (91).

Another key feature of humoral immunity is the complement system, which is involved in the chemotaxis of hemocytes to the site of infection, the opsonization of bacteria and their lysis after the assembly of the membrane attack complex (MAC), which disrupts the intruder’s membrane. MAC assembly requires the conversion (activation) of the complement component C3 to C3b, through the mannan-binding lectin (MBL) pathway (92). Also, a C1q-like protein, which is seldom found in gastropods (91), has been found in the proteome of *P. canaliculata* hemocytes (57). Recently Gorbushin, in a phylogenomic study that included *P. canaliculata* (93), has proposed a model for the evolution of an immune complex that predicts the occurrence of multiple complement-like systems in different animal taxa, and a proto-complement complex is indeed highly conserved in molluscs (93). The recognition elements MBLs and FCNs (ficolins) have not been identified in the Lophotrochozoa; however, CLECT-related proteins (CREPs), containing one or two immunoglobulin-like motifs in the N-terminal, are present in both Heterobranchia and Caenogastropoda (91).

Besides complement, other important soluble immune effectors are the antimicrobial peptides (AMPs) (94, 95) that according to the Antimicrobial Peptide Database (APD; http://aps.unmc.edu) have been mostly found in amphibians, mammals and insects and, are much less numerous in gastropods. However, some have been reported in bivalves (96, 97). In Ampullariidae, to our knowledge, there has been a single report in *Pomacea poeyana* (98).

### 4.2 The Hematopoietic System

#### 4.2.1 The Initial Studies

The first consideration of a reno-pericardial origin of gastropod hemocytes was likely that of Perrier (58), based on his histological findings in the caenogastropod *L. littorea*. However, confronting views were raised by Cuénot (59) who held that hemocytes in a variety of invertebrates could originate in all mesenchymatous tissues and the hemocoel. Same opinions were held by Haughton (99) and, much later, by Sminia (52), in a detailed study of *Lymnaea stagnalis* that included light and electron microscopy.

In turn, Pan (5) proposed again the existence of a specific site for hematopoiesis in non-infected *B. glabrata* snails, and he localized it in the blood sinuses of the pericardial wall, and in the connective tissue surrounding the kidney (p. 262). This was in agreement with the constitutive hemocyte aggregations we defined in section **3**. Also, he reported “an extreme hyperplasy of this tissue in pathological conditions”, as well as hemocyte infiltration in the connective tissue of other regions, particularly in the rectal ridge (p. 263), which was also in agreement with the distinction between constitutive and contingent hemocyte aggregations and with our evo-devo hypothesis.

Later, however, Lie and coworkers (7, 8) raised some objections regarding the precise location of the hemocyte aggregations, which according to him were present in the reno-pericardial territory but also in contact with the mantle cavity. Lie named these hemocyte aggregations the “amebocyte-producing organ”, or APO (100, 101), a designation that was to become popular.

#### 4.2.2 Defining Hematopoietic Tissues Through Challenges and Responses

Many hemocytes die as a consequence of their defensive functions and therefore a continuous generation (hematopoiesis) is needed for homeostasis. This need increases as a consequence of active infections or in other situations in which blood cells die massively, and hematopoietic cells should have the ability to proliferate in response to these challenges. Therefore, the definition of hematopoietic cells, tissues, or organs has made use of different procedures to elicit the cellular reactions of hematopoiesis. In gastropods, the procedures included from the mere exposure to potential intruders in the aquaria to the injection of parasite larvae, microorganisms, or their components (e.g., liposaccharides, LPS; 13, 29, 65, 102). Other approaches have been blood withdrawal or the pharmacological elimination of blood cells, which were also expected to elicit the hematopoietic reaction/s (62, 103).

An accurate definition of a hematopoietic site should rely not only on precising the challenge used, but also on refining the procedures for detecting the mitotic activity and quantifying it during homeostasis and in response to a challenge. For many years, hematopoietic responses were non-quantitatively assessed through the mere observation of cellular reaction/s to intruders, mainly helminth parasites (e.g., 5, 7, 8). Later, however, measurement of the mitotic index was introduced (e.g., 104–107) and this was further refined by the immunodetection of markers of cell division, such as bromodeoxyuridine, a synthetic nucleoside that is incorporated in place of thymidine during the S-phase of DNA replication (e.g., 29). Other markers have been used which are directed to detect different phases of the cell cycle, but that may have drawbacks, as detecting the constitutive expression of their respective targets (e.g., 108). Another approach has been to detect not the mitotic activity itself but the consequences of it, e.g., by labeling cytoplasmic proteins and detecting their dilution by the successive cell divisions (32). A related approach may be the measurement of the size changes of the presumptive proliferative site, although it should be kept in mind that these measurements may also be affected by the attraction of hemocytes to the particular site (29). Since all methods may have drawbacks, the concurrent use of more than one method seems advisable.

#### 4.2.3 Hematopoiesis in the Ampullariidae

##### 4.2.3.1 A Pending Question of Anatomical Nomenclature

There is an old issue of anatomical nomenclature that has caused much confusion, and that it is still causing troubles in recent literature. In a way, the question may be posed as simply as such: “How many kidneys do the ampullariids have?” And the right answer to the question may also be simple: “Just one, as all caenogastropods do”. Therefore, one can ask, “Why there are so many references in recent literature to an “anterior” and a “posterior kidney” in Ampullariidae? This needs to be clarified because the kidney is important as a potential hematopoietic organ in this family and the confusion is growing (see section **4.2.3.3**).

The confusion originated in Bouvier’s and Perrier’s writings (58, 109), at the end of the 19th century. Perrier was involved in a discussion on the fate of the right renal primordium in adult “monotocardian”, while Bouvier, who was himself a pioneer of ampullariids anatomy, had wrongly interpreted that what is now recognized as an ectodermal ureter (25, 26) was indeed a derivative of the right renal primordium, and therefore it was a true kidney (109). Bouvier called it “anterior kidney”, as opposed to the kidney proper, which he called the “posterior kidney”. And because both “kidneys” were connected by a “nephrostome”, Perrier became convinced (p. 171) that the ampullariids represented an intermediate case between the two-kidney condition of *Patella* and the single kidney of other “monotocardians”, and that it was *un passage intermédiaire* (a “missing link”)! supporting his interpretation that a complete fusion of both primordia originated the single kidney of “monotocardian” gastropods.

Even though Perrier’s (58) interpretation did not gain general acceptance and was finally forgotten, his paper contributed to make better known the views of Bouvier, and the names of anterior and posterior kidneys did survive for more than a century and continue to cause difficulties today. Formally, the confusion was brought to an end by Andrews in 1981 (24), when she introduced the term “ureter” for the Ampullariidae, and referred to it as “the so-called anterior kidney of the earlier authors”. Those “earlier authors” included even herself (110), and we have also used such terminology once (63). Also, the malacological community assimilated slowly her formal corrigendum and, until recently, many continued to fall into the two-kidney confusion (e.g., 62, 65, 111). We should emphasize that these organs are morphologically (29), developmentally (25), and functionally (24) different, and that confounding them has had significant negative consequences (see section **4.2.3.3**).

##### 4.2.3.2 Do Ampullariids Have Hematopoietic Site/s?

Yousif et al. (13) first suggested the occurrence of an “amoebocyte-producing organ” in an the ampullariid *M. cornuarietis.* The proposed site was a cellular band extending along the lung roof that became markedly thickened in snails infected with *A. cantonensis* larvae. This report seemed to disprove our evo-devo hypothesis that restricted the constitutive hematopoietic sites to the reno-pericardial complex, and therefore we carefully looked for an equivalent tissue in the lung of the only ampullariid we had at hand, *P. canaliculata*, but we did not find any such tissue in serially sectioned lungs, neither in the roof nor in any other part of the lung (112).

Another study that was made in *P. canaliculata* (62) had proposed the existence of a hematopoietic tissue within the pericardial cavity, more precisely in the external aspect of the “veins” entering the “heart”, as well as in the pericardial fluid. The same idea was put forward in a collaborative chapter (113) and the study was referred to in an influential recent review of hematopoiesis (21). We think it is erroneous, however. For untangling this matter, it should be first said that the “veins” shown in micrographs of both (62) and (113) were actually the auricle, that shows a thin wall and characteristic muscular bundles traversing its lumen, as it was described by Andrews (110; p. 83), while the referred “heart” was in fact the ventricle, whose connection with the single auricle is shown in Figure 2B of (62) and microscopically in Figure 1.1 of (113). It should also be said that the mitotic marker used, phospho-Ser10 histone H3, may not be specific, because it also marks non-dividing cells, such as neurons (108) in *P. canaliculata*. Notably, a large amount of free hemocytes in the pericardial fluid was shown (62; their Figure 6), all of which were marked with anti-phospho-Ser10 histone H3, both in control and challenged animals. Furthermore, even though no quantitation was attempted, the authors stated their number was not affected by blood withdrawal, i.e., the hematopoietic challenge they used (62; legend to their Figure 6). Also, some large nuclei were marked in the pericardial fluid, which were larger than those of hemocytes (shown in their Figures 6D–F but not mentioned in the legend). An important hint for the identification of the latter cells came indeed from (62; their Figure 5), and from (113; their Figure 1.1) where these cells appear orderly arranged in the epicardial epithelium of the auricle, indicating these marked epithelial cells were not hemocytes but the larger “epicardial cells” described by Andrews (114) in *M. cornuarietis*. Besides that, it should be said that there is no “pericardial side” of the pulmo-branchial and renal veins, because they are embedded in connective tissue before debouching into the auricle and do not have an intrapericardial path (110, 115). Epicardial cells are functionally involved in ultrafiltration and hence in the generation of the primary urine (22, 114), and they seem to constitutively express phospho-Ser10 histone H3, as some neurons of *P. canaliculata* may also express (108). Therefore, there is no evidence of a hematopoietic response to the experimental challenge used, and we should conclude that there is no empirical indication of a hematopoietic response in the pericardium of *P. canaliculata*, neither in the pericardial fluid nor in its epithelial lining.

Besides that, as it has been pointed out elsewhere (29), a potential hematopoietic site should have a direct connection with the hemocoel, where the newly generated hemocytes should pass, while the pericardial cavity connects not with the hemocoel but with the renal cavity and, if hemocytes were generated there, they would get finally expelled with urine. All previous propositions of hematopoietic sites in the pericardium of other species were in the hemocoelic sinuses that are contained within *the pericardial wall* (i.e., not in *the pericardial cavity*), because only those hemocoelic sinuses warrant a direct access to the systemic circulation.

Therefore, we had to look in a different direction and, according to our evo-devo hypothesis, we put to test the possible hematopoietic role of the only constitutive hemocyte aggregation we had noticed in *P. canaliculata*, i.e., the renal hemocyte islets. To test this possibility, the animals were injected with yeasts in the visceral mass (or with buffer) and cell proliferation in the islets was compared by (1) histological examination, (2) a bromodeoxyuridine incorporation assay, and (3) by measuring the changes in the renal area occupied by the hemocyte aggregates. All these different approaches were concurrent in indicating a hematopoietic response to the immune challenge: (1) nodules were formed in the hemocyte islets (**Figure 7**), and (2) both bromodeoxyuridine incorporation and the area occupied by the hemocyte aggregates increased significantly (29). This was the first quantitative demonstration of a hematopoietic response to an immune challenge in an ampullariid and, to our knowledge, in the wide diversity of the Caenogastropoda.

However, we may still wonder whether the ampullariids have a hematopoietic organ in the pericardial blood sinuses, which certainly have direct passages to the hemocoel. This would be homologous to the APOs shown in several lymnaeoid and helicoid snails. However, this remains to be explored.

##### 4.2.3.3 A Prokineticin-Like Protein as a Potential Cytokine Promoting Hematopoiesis

There is much to be learned about the molecules and mechanisms that guide the hemocyte proliferation and differentiation in gastropods (21), and especially in ampullariids. Therefore, we were much interested when we knew of the proposition of a cytokine-candidate that would increase hemocyte proliferation in *P. canaliculata* (65). It was molecularly related to the prokineticin-domain containing proteins, astakines-1 and -2, which were discovered in the crayfish *Pacifastacus leniusculus* (116). Astakines promote the release of new hemocytes from the hematopoietic tissue of this crayfish and induces cell division and the partial cell differentiation of hematopoietic cells in primary culture (117). Similar results were obtained in the shrimp *Penaeus monodon* (118). Also, a polypeptide containing a homologous sequence (named CgAstakine) was identified and found active in the Pacific oyster, *Crassostrea gigas* (119). In *P. canaliculata*, the cytokine-candidate was provisionally named Pc-PlP (for *P. canaliculata*-prokineticin like protein) (65), and changes in the expression of the *Pc-PlP* gene were compared with that of the housekeeping gene *rpL5* in several tissues/organs, both in control (constitutive) conditions and after two different challenges, repeated blood withdrawals and LPS injection. The results obtained were not easy to interpret and the authors attempted to correlate them with propositions made in previous publications and with which we should dissent, as it has been previously discussed in this review. Those propositions were that the aortic ampulla was a hemocyte reservoir and a site for hemocyte maturation (reasons discussed in section **4.1.3.2**) and that the pericardial cavity and its fluid were sites of hematopoiesis (reasons discussed in section **4.2.3.2**).

Also, the paper considers the ureter (i.e., the “anterior kidney” of the older terminology, see section **4.2.3.1** for the source of this confusion) as the most responsive organ to LPS injection but, while attempting to relate their findings with hematopoiesis, the ureter is referred to as “the rostral component of an organ containing islets of hemocytes and proposed to be an immune barrier (Cueto et al., 2015)”, i.e., that the ureter and the kidney were mixed-up. Indeed, the immune barriers we proposed were the actual kidney and the lung (29), and the hemocyte islets were found only in the kidney (41), not in the ureter (see section **4.2.3.2**). In fact, the ureter is not the rostral component of the kidney (24), it is a quite different organ (see section **4.2.3.1**). In the same context, the occurrence of commensal microorganisms in the kidney was surprisingly attributed to a paper by us (i.e., 41), in which we said nothing regarding commensal microorganisms. The paper (65) also has some other inaccuracies regarding sampling and organ identification. One of them is that some intrapericardial “vessels” were sampled, which cannot be vessels but the auricle (as already explained in section **4.2.3.2** together with the consequences of this misidentification).

In our view, the reported pattern of control and challenged *PcPlP* expression does not suggest any clear role for this prokineticin-containing protein in the regulation of hematopoiesis in *P. canaliculata*. However, all the objections above should not obscure the interesting finding of a protein that bears structural similarities with the astakines that promote hematopoiesis in two crustaceans (116–118) and a bivalve (119) and that is encoded in the *P. canaliculata* genome (20). Though this is interesting in itself, we still have to learn about the actual function of this protein in *P. canaliculata*, which may or may not be related to hematopoiesis or to any other function of the immune system.

#### 4.2.4 Hemocyanin and Its Synthesis and Polymerization

##### 4.2.4.1 The Peculiarities of one of the Largest Proteins in Nature

Hemocyanin is the most widespread respiratory pigment in molluscs, though it is lacking in the Patellogastropoda (120) and the Planorbidae, where it is replaced by hemoglobin (121). Sminia (52) was the first to observe polymerized hemocyanin in rhogocytes (also called “pore cells”, see section **4.2.4.2**) of *L. stagnalis* and to propose that this pigment was synthesized and/or stored in these cells. More recently, Martin et al. (122) and Kokkinopoulou et al. (123) have also shown evidence for the localization of hemocyanin synthesis in rhogocytes, but no ultrastructural evidence was obtained for *P. canaliculata* (64, 112). The size and structure of hemocyanins vary among different taxa and are among the largest proteins in nature, with molecular masses ranging from 3.5 to above 13.5 MDa (124) and are thus visible under the transmission electron microscope (in *P. canaliculata*: 29, 125, 126). Hemocyanins have biomedical importance, and the first to be studied was that of the keyhole limpet (*Megathura crenulata*) which has been used for the specific treatment of bladder carcinoma (its efficacy probably due to a cross-reacting carbohydrate epitope) and is also widely used as a hapten carrier and generalized vaccine component (127). However, the hemocyanin of at least four other gastropod species have been studied: *Concholepas concholepas*, *Haliotis tuberculata*, *Rapana thomasiana* and *Fissurella latimarginata* (128).

The hemocyanin of *P. canaliculata* has received particular attention in recent proteomic and genomic studies. It is a multimeric protein encoded by four similar but sequentially distinct genes (126). Mining in the known genomes and transcriptomes of other three ampullariid species (*Marisa cornuarietis*, *Pomacea maculata*, and *Lanistes nyassanus*) (20, 129) indicates that the four different subunit isoforms seem typical of the family. Those subunits are polymerized forming di-, tri- and tetradecamers (being di- and tri-decamers more abundant than tetradecamers) which could be seen under TEM.

##### 4.2.4.2 Rhogocytes: Large and Polyploid Hemocytes?

These cells are thought to be exclusive of molluscs and may be the only distinct character exhibited by all extant species of the phylum (130, 131). A distinctive morphological character of these cell is a “slit apparatus” occurring in the plasma membrane as the outer limit of “extracellular lacunae” (=“subsurface cisternae”; 132) and that may serve as a molecular sieve (see below) (121, 123). They are usually embedded in the connective tissue, as resident hemocytes are, but free-floating rhogocytes have also been mentioned in the hemocoel (e.g., 133, 134). Haszprunar (130) proposed the homology of rhogocytes with podocytes, nephrocytes, and cyrtocytes, which links these cells with the excretory system in molluscs and some non-molluscan phyla, and thereby with the gastropod reno-pericardial system and with our evo-devo hypothesis.

**Figure 10:**
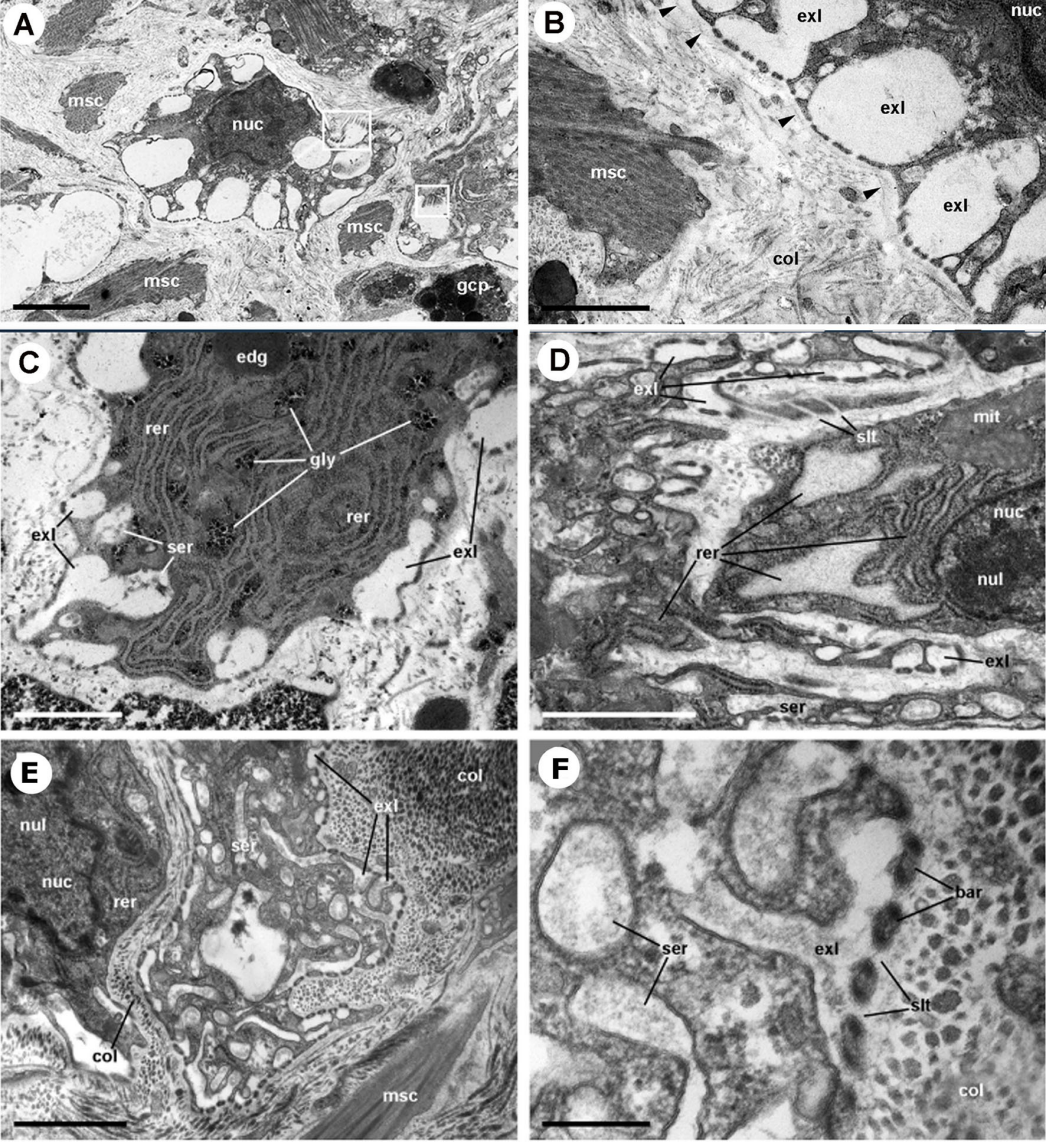
Rhogocytes in the lung (under TEM). **(A)** A rhogocyte and the cytoplasmic extension of another one, showing the extracellular lacunae with the characteristic “slit apparatus”; they are surrounded by a lamina densa and extracellular matrix where muscle fibers and storage cells are embedded. Most components of the slit apparatus (i.e., bars and slits) appear sectioned transversally, but the two dashed boxes show some longitudinally sectioned. Scale bar represents 2 µm. **(B)** Detail of the extracellular lacunae of the same rhogocyte. Arrowheads indicate the extracellular matrix that surrounds the rhogocyte. Scale bar represents 1 µm. **(C)** Rough endoplasmic reticulum with interspersed glycogen clumps; profiles of the smooth endoplasmic reticulum are also seen in the vicinity of the extracellular lacunae. **(D)** Wide cisternae of the rough endoplasmic reticulum, and a large nucleolus within an oval nucleus; cytoplasmic extensions found in the vicinity (probably from other rhogocytes) contain rough endoplasmic reticulum and extracellular lacunae. **(E)** A cytoplasmic region showing a wide cistern of the smooth endoplasmic reticulum as wells as multiple tubuli, some of which connect with extracellular lacunae. **(F)** Close-up of a connection of the smooth endoplasmic reticulum with an extracellular lacuna; the slit apparatus opens to a region of packed collagen fibers. BAR, bar of the slit apparatus; COL, collagen matrix; EDG, electron-dense globule; EXL, extracellular lacunae; GCP, glial cell process; GLY, glycogen; MIT, mitochondria; MSC, muscle cell; NUC, nucleus; NUL, nucleolus; RER, rough endoplasmic reticulum; SER, smooth endoplasmic reticulum; SLT, slit of the slit apparatus. Scale bars represent: **(A)** 2 µm; **(B–D)** 1 µm; **(E)** 500 nm; **(F)** 200 nm. From (112).

Rhogocytes in *P. canaliculata* **(** **Figure 10** **)** show large, pleomorphic, and frequently notched nuclei, containing heavy heterochromatic clumps and conspicuous nucleoli. The tubuli and cisternae of both the rough (RER) and smooth (SER) endoplasmic reticulum are frequently dilated and filled of a uniform material with an electron density similar to that of the surrounding cytoplasm, but there are no distinctive signs of phagocytic activity. Another feature of rhogocytes are the cytoplasmic granules of varying electron density, that may become very large, likely by the merging of smaller ones.Taken together, these nuclear and cytoplasmic features would suggest polyploidy and a high level of transcription and post-transcriptional processing.

**Figure 11:**
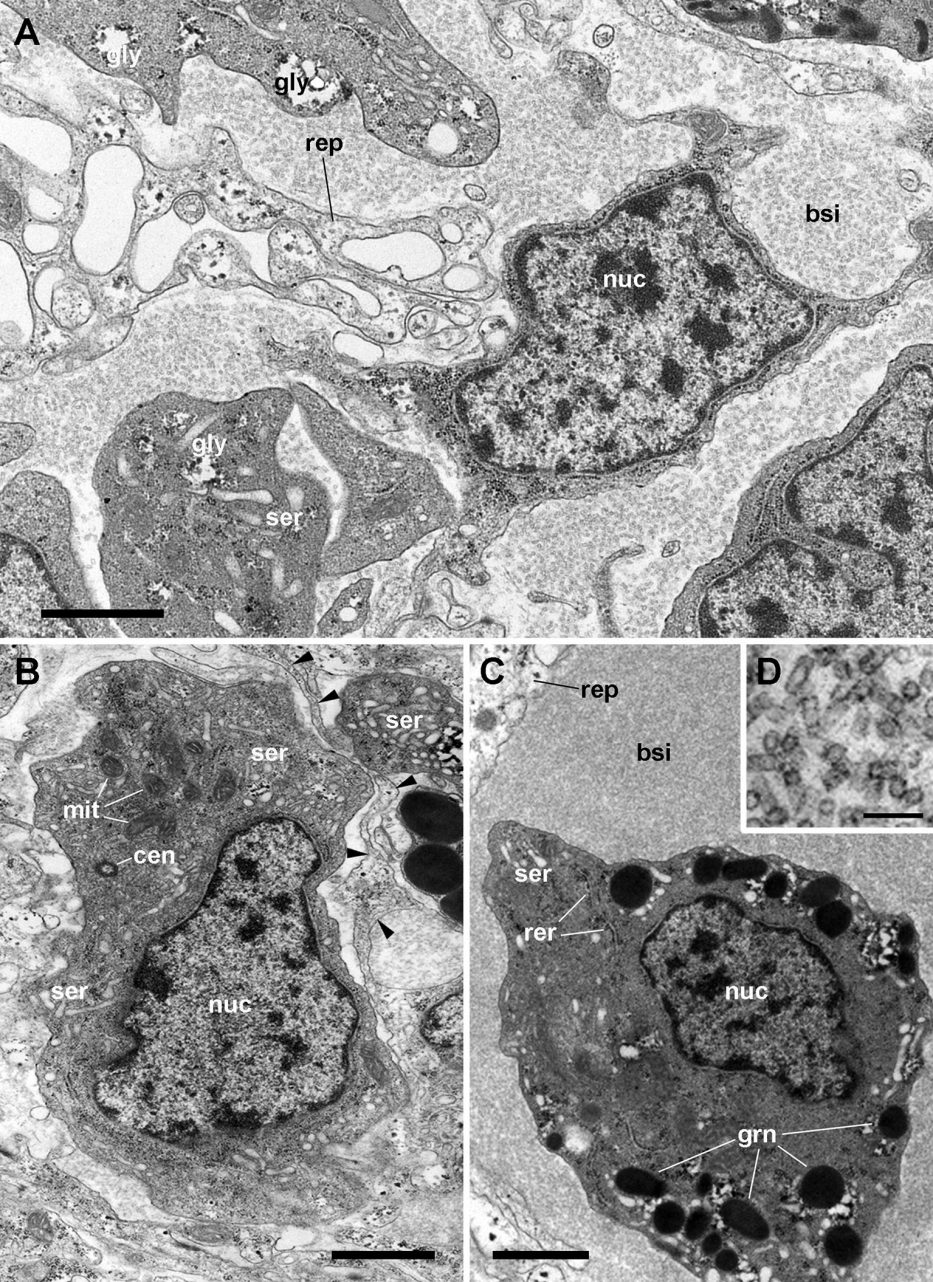
Renal blood sinuses, hemocytes, and hemocyanin polymers under TEM. **(A)** Blood sinus containing hemocyanin polymers and an agranulocyte lying beside a projection of a renal epithelial cell. Parts of other hemocytes and of the epithelial extensions show dark clumps and non-membrane bound areas, which are likely glycogen deposits. **(B)** A hyalinocyte showing eccentric nucleus, profiles of the SER, and numerous mitochondria. Intimate contacts between different hemocytes and thin epithelial projections are seen (arrowheads), but there are no intercellular junctions. **(C)** A hemocyanin filled blood sinus containing a granulocyte with an eccentric nucleus and electron-dense R-granules. **(D)** Detail of hemocyanin polymers. Scale bars represent: **(A–C)** 1 µm; **(D)** 100 nm. BLA, basal lamina; BSI, blood sinus; CEN, centriole; grn, R-granules; MIT, mitochondrion; NUC, cell nucleus; REP, renal epithelium; RER, rough endoplasmic reticulum; SER, smooth endoplasmic reticulum. From (29).

Regarding hemocyanin synthesis, it is intriguing that *P. canaliculata* rhogocytes do not show the typical hemocyanin polymers seen in some gastropod species (e.g., 52, 122, 123) while loads of hemocyanin polymers may occur in the blood spaces around the renal hemocyte islets of this species (**Figure 11**) (29). Could it be possible that hemocyanin subunits were synthesized in rhogocytes, and transported to the kidney for polymerization?

**Figure 12:**
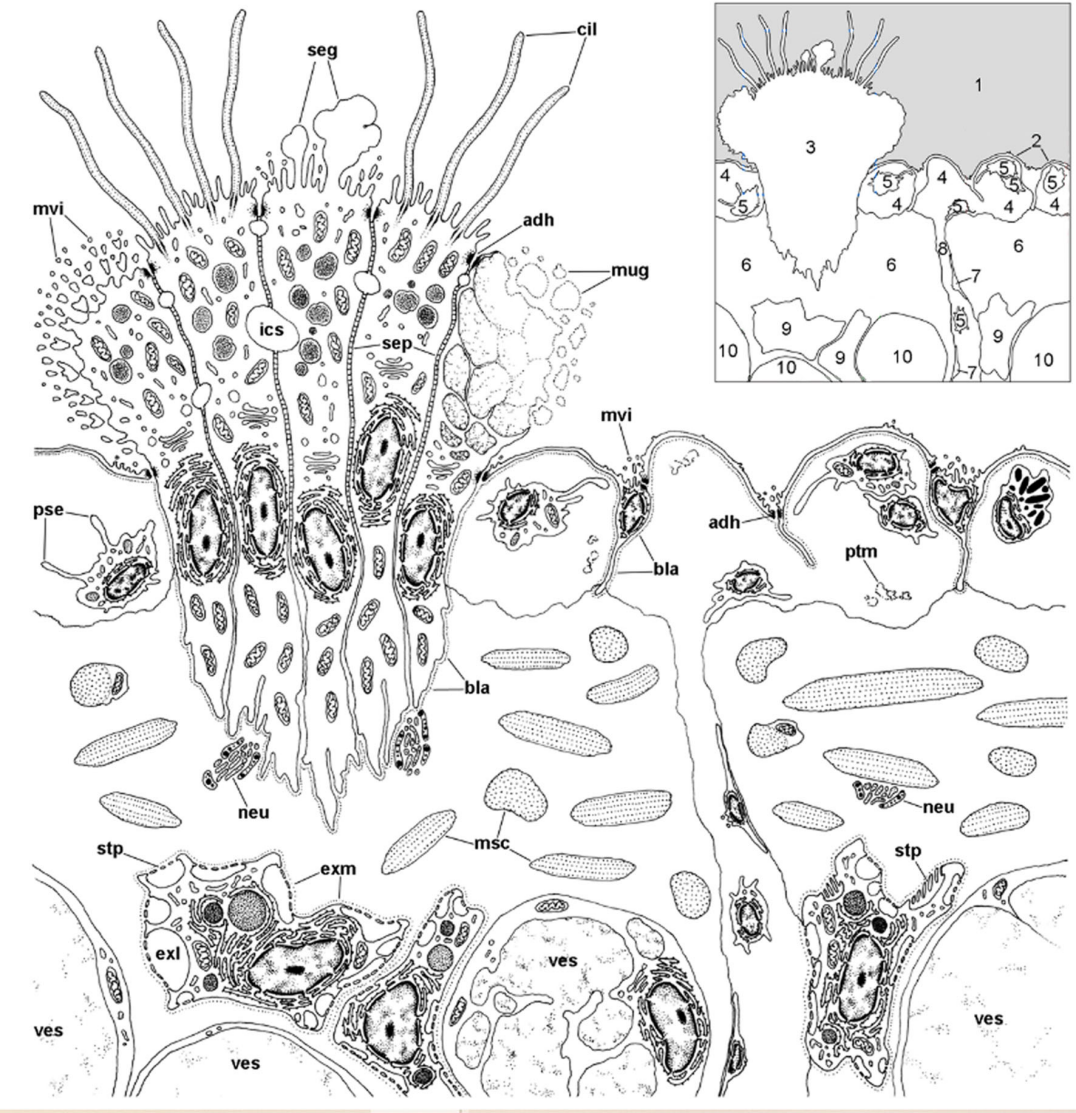
Location of rhogocytes in the lung (illustration credit: Guido I. Prieto). The main structures are indicated in the thumbnail in the right-upper corner. The diagram depicts a portion of the respiratory lamina (with a ciliary tuft and some blood sinuses), the fibromuscular layer that underlies it (the collagen matrix of the fibromuscular tissue has not been drawn for clarity) and the upper part of the storage tissue layer. Rhogocytes mainly occur in contact with storage cells, but also with the perpendicular blood sinuses that feed the respiratory lamina and, less frequently, are found in the proximity of the basal part of the ciliary tufts (not shown in the diagram). Rhogocytes are encased in a thin lamina of extracellular matrix (dashed lines). 1, pulmonary cavity; 2, pavement cells; 3, ciliary tuft; 4, blood sinuses of the respiratory lamina; 5, hemocyte; 6, fibromuscular tissue; 7, endothelial-like cell; 8, radial sinus; 9, rhogocyte; 10, storage cell; ADH, adherent junction; BLA, basal lamina; CIL, cilia; EXL, extracellular lacunae; EXM, extracellular matrix; ICS, intercellular space; MSC, muscle cells; MUG, mucin granules; MVI, microvilli; NEU, neurite bundle; PSE, pseudopodia; PTM, particulate material; SEG, secretory globules; SEP, septate junctions; STP, slit apparatus; VES, vesicles of storage cells. From (109).

We first found rhogocytes in the lung of *P. canaliculata* at the interface between the fibromuscular layer that underlies the respiratory lamina and the storage tissue that forms most of the pulmonary wall in this species (112) (**Figure 12**). These rhogocytes were in close association with the storage cells that accumulate urates (63, 135) and possibly glycogen (136) in this species. The distribution of these storing cells in ampullariid tissues is well known (63), but not that of rhogocytes, that have only been searched for in the lung and the aortic ampulla (64, 112).

##### 4.2.4.3 Storage Tissues and Their Association With Rhogocytes

Cells with storing capacity for quite different substances have received different names in gastropods (e.g., vesicular cells, globular cells, chondroid cells, plasmatic cells, Leydig cells, etc.) and were frequently associated with glycogen storage (22, 28). Because the phylum Mollusca lacks a specialized tissue as the adipose tissue of vertebrates (137), glycogen storing cells and tissues may be serving an analogous role in energy metabolism. However, other substances are stored in these tissues in *P. canaliculata* and other gastropod taxa [such as calcium carbonate in the congeneric *P. maculata* (138), or copper in a likely homologous crystalloid-containing tissue in *L. littorea* (139)].

A functional interpretation of the close association of rhogocytes with cells of the storage tissue of *P. canaliculata* (63, 64, 112) is wanting. Uric acid is accumulated intracellularly in this tissue in the form of spheroidal crystalloids (135). This purine, which has been generally considered an excretory compound, circulates in plasma at a concentration of ~5 mg/L (140) and is totally resorbed from the primary urine, so that it is not excreted (135). The storage cells are integrated in a stress-related tissue system that is anatomically discontinuous because it is present in some specific organs only (mainly the lung, the digestive gland, the wall of the style sac, the mesenterium of the coiled gut, the testis and the heart ampulla) (63). Physiological studies have shown uric acid released from these sites participates in the adaptive responses to oxidative stress when the animal enters or leaves the hypometabolic states of estivation and hibernation (141, 142), and proteomic studies have confirmed they also partake in other adaptations to stress (hypoxia or pesticide exposure) (64, 143).

## 5 Main Threads Interwoven in Our Synthesis (Not Necessarily in Chronological Order)

(a)In the primeval years of Biology as an independent discipline, Lamarck identified the main taxa we have been studying for more than two hundred years after him.
(b)In this review, we hypothetically associated the origin of the gastropod immune system with that of the reno-pericardial system.
(c)We distinguished constitutive hemocyte aggregations from those that are contingent upon the occurrence of an immune challenge, and we hypothetically associated with the constitutive aggregations some hematopoietically committed progenitor cells, which may spread from the constitutive centers and originate contingent aggregations in other places.
(d)Though contingent aggregations can occur anywhere in the body, they are likely facilitated in places where the circulation stagnates and the contact of hemocytes with the intruders becomes more likely. In *P. canaliculata*, we found that the kidney, that has constitutive hemocyte aggregations, and the lung that has none, both act as immune barriers, because of they are critically interposed in the circulation.
(e)Three different populations of hemocytes (hyalinocytes, granulocytes, and agranulocytes) were characterized in the circulation of *P. canaliculata*, as well as after dissociation of renal hemocyte islets. Image3C, a novel, high-throughput, image-based method, may revolutionize studies of these cells in the near future.
(f)Phagocytic activity was found in circulating hemocytes and in those recovered from dissociations of renal hemocyte islets; in both cases, the phagocytic index was higher in the hyalinocyte population.
(g)A marked tendency of *P. canaliculata* hemocytes to form spheroidal aggregates was discovered in axenic primary cultures (similar to the nodules formed *in vivo* in the kidney and lung after immune challenging). Hemocyte aggregations in axenic cultures should be elicited by a contact factor.
(h)Our evo-devo hypothesis predicted possible proliferative sites in constitutive hemocyte aggregations in the reno-pericardial territory and, to test this, we measured the incorporation of Br-deoxyuridine in the renal hemocyte islets as well as the size of the renal islets. We found both parameters increased after an immune challenge (yeast injection in the hemocoel).
(i) Repeated blood withdrawals did not produce a marked drop in circulating hemocytes indicating that hemocyte reservoir/s should exist.
(j)The renal hemocyte islets are postulated as hemocyte reservoirs because of their size, their loose architecture (which facilitates the in-and-out hemocyte movements), as well as the hematopoietic ability of their cells to restore the hemocyte numbers within the reservoir, and in the systemic circulation.
(k)The proliferative capacity of the hemocyte islets has been quantitatively shown and, to our knowledge, it is the sole quantitative demonstration of tissue hematopoiesis in the Caenogastropoda.
(l) Soluble immune effectors have been less studied than in other gastropod taxa. They include lectins (some in the albumen gland or within eggs, where they may protect the embryo) and phylogenomic studies have also indicated a presumptive “primitive complement system” in the Apogastropoda (i.e., Caenogastropoda plus Heterobranchia), thus including ampullariids. Information on antimicrobial peptides is even scarcer, and we have only known of a single compound reported in a *Pomacea* species.
(m)The complete gene structure of hemocyanin, the respiratory pigment, has been recently described in *P. canaliculata* and it is the only one known among the Caenogastropoda

## 6 The Provisional Limits

We have gone through more than two centuries of history, guided by our evo-devo hypothesis, and the journey is coming to an end. These are but the provisional limits of our understanding of ampullariid immunity. They present themselves to us as a tightly woven fabric, though not without holes of controversy, and loose edges of speculation. As a research group, or even on a personal level, we can look back over at least the last two decades and say, “How ignorant we were and how much we have learned from these animals.” Fortunately, there is still much to be done.

## Author Contributions

Conceptualization: Investigation: CR, IV, and AC-V. Writing: CR, IV, and AC-V. Supervision: AC-V. Project Administration: IV. Funding Acquisition: CR, IV, and AC-V. All authors contributed to the article and approved the submitted version.

## Funding

The work in the authors’ laboratory during the last years has been supported by grants from Universidad Nacional de Cuyo, Consejo Nacional de Investigaciones Científicas y Técnicas, and Agencia Nacional de Promoción de la Investigación, el Desarrollo Tecnológico y la Innovación (PICT 2019-03211).

## Conflict of Interest

The authors declare that the research was conducted in the absence of any commercial or financial relationships that could be construed as a potential conflict of interest.

## Publisher’s Note

All claims expressed in this article are solely those of the authors and do not necessarily represent those of their affiliated organizations, or those of the publisher, the editors and the reviewers. Any product that may be evaluated in this article, or claim that may be made by its manufacturer, is not guaranteed or endorsed by the publisher.

## Footnotes

1 All taxonomic names of superfamilial or higher-level ranks are referred according to Bouchet et al. (3). Names of familial or lower-level ranks follow the last available revision of the particular taxon, usually as they are referred to in WoRMS (marinespecies.org).
